# Developing an Online Community Advisory Board (CAB) of Parents From Social Media to Co-Design an Human Papillomavirus Vaccine Intervention: Participatory Research Study

**DOI:** 10.2196/65986

**Published:** 2025-04-16

**Authors:** Regan M Murray, Shawn C Chiang, Ann C Klassen, Jennifer A Manganello, Amy E Leader, Wen-Juo Lo, Philip M Massey

**Affiliations:** 1 Department of Mental Health, Johns Hopkins Bloomberg School of Public Health, Baltimore, MD, United States; 2 Department of Health Behavior, Texas A&M University School of Public Health, College Station, TX, United States; 3 Department of Community Health and Prevention, Drexel University, Philadelphia, PA, United States; 4 Department of Health Policy, Management and Behavior, University at Albany, State University of New York, Albany, NY, United States; 5 Division of Population Science, Department of Medical Oncology, Thomas Jefferson University, Philadelphia, PA, United States; 6 Department of Counseling, Leadership, and Research Methods, University of Arkansas, Fayetteville, AR, United States; 7 Department of Community Health Sciences, University of California, Los Angeles, 650 Charles E Young Drive S, 4th Fl, Los Angeles, CA, 90095, United States, 1 424-259-5258

**Keywords:** online community advisory boards, community engagement, social media, digital health, digital health intervention, HPV vaccine, human papillomavirus, HPV, parent health, child health

## Abstract

**Background:**

Social media health interventions have grown significantly in recent years. However, researchers are still developing innovative methods to meaningfully engage online communities to inform research activities. Little has been documented describing this approach of using online community advisory boards (CABs) to co-create health communication interventions on social media.

**Objective:**

This study describes the formation, engagement, and maintenance of an online CAB focused on co-creating a health education intervention for parents regarding the human papillomavirus (HPV) vaccine. The study provides guiding principles for public health researchers implementing such CABs in future digital health interventions.

**Methods:**

In May 2020, Twitter was used to recruit parents of children aged 9‐14 years, who were active users of the platform and were interested in serving on a CAB focused on child health and online programs. The recruitment campaign included Twitter (rebranded as X in 2023) advertising tools (eg, “interests” and “audience look-a-likes”). A total of 17 parents completed a screening survey and 6 completed a follow-up phone interview. Following phone interviews, 6 parents were invited to join the CAB, where they committed to a 1-year involvement. The CAB participated in eleven 1-hour online meetings in the first year, contributing to monthly feedback through participatory workbooks. Long-term engagement was sustained through icebreakers and casual online interactions, as well as providing real-time updates to demonstrate CAB feedback integration. An anonymous midterm evaluation was conducted at the end of the project’s first year to assess processes and identify future growth opportunities.

**Results:**

A total of 6 parents (5 females and 1 male) with children aged 9-14 years from diverse racial and ethnic backgrounds (African American, South Asian American, and White) across 6 states in the United States, representing urban, suburban, and rural areas, agreed to serve as CAB members. All 6 CAB members committed to 1 year of service beginning in July 2020 with 4 extending their participation into a second year (August 2021-August 2022). The CAB provided expert insights and feedback to co-develop the intervention, including character development, narrative content creation, study recruitment, survey development, and intervention delivery. The midterm evaluation showed 100% (6/6) satisfaction among CAB members, who valued the connections with other parents and their contribution to research. While all members felt confident discussing HPV, 83% (5/6) suggested diversifying the group and increasing informal bonding to enhance engagement and inclusivity, especially for differing vaccination views.

**Conclusions:**

This study demonstrates that online CABs are a highly effective model for co-creating and informing online health communication interventions. The engagement of parents from diverse backgrounds and the structured use of online tools (eg, interactive workbooks) creates a constructive and thoughtful environment for incorporating parent contributions to research. This study highlights guiding principles to forming, engaging, and maintaining an online CAB to enhance health research and practice.

## Introduction

Community advisory boards (CABs) are a widely integrated methodology in participatory health research to understand community needs, advise research, and ultimately improve health behaviors or health outcomes [1]. Comprising community members, leaders, stakeholders, and others, CABs are united by a common health interest, identity, status, or experience [2]. In the field of public health, CABs are integral to research programs to provide unique perspectives and guidance related to proposed research questions and interventions. Researchers often implement participatory strategies and inspire innovative discussions that lead to novel approaches to address health-related issues that are aligned with community needs [3]. Over time, as community needs evolve and change, CABs can also shape the development and adaptation of interventions to better align with those identified needs. Engaging community members early and often in the research process is an effective strategy to increase efficacy of the designed intervention, enhance transparency, and reduce population-level disparities [4].

In the context of child health, advisory boards consisting of parents or caregivers are essential for ensuring that public health research and interventions are aligned with family values and needs [5-7]. CABs comprising parents and caregivers have addressed a wide range of public health topics including but not limited to asthma [8], Early Head Start programs [9], childhood obesity [10], and childhood vaccination [11]. For parents, opportunities to engage in research focused on child health have been shown to be driven by altruistic motivations, previous experience in research or health care, and desire to share personal perspectives [12]. Parents are often the ultimate decision makers when it comes to their child’s health; incorporating their input and understanding their perspectives provides important insights into facilitating the adoption of health behaviors among children.

As public health research and practice are incorporated more into the social media and online environment, the need for online CABs is essential. For parents, online and internet-delivered health interventions targeting child health continue to grow [13-15] and CABs will be an important approach to engage and work alongside diverse communities. Already, online opportunities to engage parents in research have allowed participation of varied perspectives across diverse geographies and facilitated ease of involvement by working around demanding parent schedules [12]. By shifting to the online environment, research teams can engage diverse populations through flexible and accessible means. Heightened flexibility allows researchers to swiftly adapt to the evolving needs of the community, as well as the ever-evolving nature of social media platforms and the online environment.

Engaging with diverse populations with varied perspectives is crucial to fostering trust, developing culturally relevant and accepted interventions, and encouraging participation in health behavior interventions [16]. This is further bolstered by expanding into the sphere of community-based participatory research through online platforms. Furthermore, as elements of everyday life increasingly transition to online platforms, the need for robust online CABs becomes paramount. This aligns with the broader trend of hybrid and remote work environments following the pandemic [17], offering flexibility that accommodates the diverse schedules and locations of members, similar to how many jobs and careers have adapted to remote work.

While the need to integrate online CABs into public health research is apparent, particularly as social media becomes an increasingly influential health intervention tool, there is a gap in evidence regarding the process of developing and maintaining online CABs. Addressing this gap is essential to promote thoughtful and systematically replicable online CABs in future public health efforts. Guiding frameworks play an important role in the development of effective CABs by providing researchers with structure and strategies for engaging CAB members from creation to evaluation [18]. Scholars interested in evaluating processes related to community-engaged advisory strategies have adapted earlier frameworks to meet the needs of systematically evaluating CABs [2]. The framework by Newman et al [2] identifies 3 primary domains that support the necessary functions of community advisory boards: formation, operation, and maintenance. Other researchers have found utility in following this guidance as well [19]. This study uses the framework developed by Newman et al [2] with some slight modifications in terminology; we refer to the “operation” domain labeled by Newman as “engagement.”

This case study contributes to limited evidence on approaches and procedures for developing online CABs. We provide a case study, focusing on the development of human papillomavirus (HPV) vaccine messages for parents who use Twitter (rebranded as X in 2023), a microblogging social media platform, to share evidence and findings to support the development of future online CAB creation. Briefly, the HPV vaccine is an important topic for child and adolescent health, and parents are critical stakeholders in deciding whether their child receives the HPV vaccine. The HPV vaccine is routinely recommended for males and females between the ages of 9 and 12 years and can also be administered up to the age of 26 years and older in some cases [20]. The vaccine protects against certain strains of HPV that are associated with various cancers, including cervical, anal, throat, and penile cancers, as well as genital warts [20]. Parent involvement in online CABs is supported by previous studies showing that the inclusion of parents in HPV vaccine communication interventions resulted in improved vaccination rates [21], decreased vaccine hesitancy [22], as well as increased education and knowledge [23].

In this study, we describe how we assembled an online CAB consisting of parents from across the United States to serve in an advisory role in the development of an online intervention. The goal of the CAB was to co-create a social media intervention on Twitter to serve as an information source for parents when deciding to get the HPV vaccine for their child. We used the framework by Newman et al [2] of formation, engagement, and maintenance to organize our Methods, Results, and Discussion sections. The purpose of this study is to describe promising strategies to be used and adapted for the creation of online CABs, ultimately to continue to support the development of participatory online health research and practice.

## Methods

### Formation

**Figure 1.**
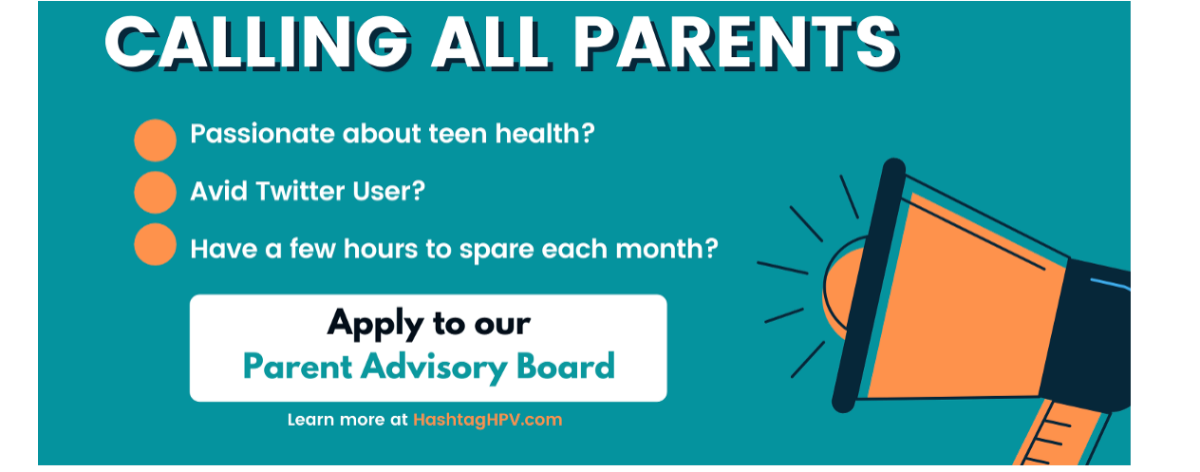
Recruitment advertisement graphic used on Twitter to recruit parents to the online community advisory board in May 2020.

To initiate the creation of the online CAB, the study team first clearly defined the purpose, roles, expected functions, and membership and recruitment strategies tailored to an online format [2]. It was determined the CAB would assume an advisory role to provide support, feedback, and review of digital materials based on the project’s needs. Given the study’s focus on developing an online digital health intervention delivered through Twitter, recruitment was purposefully conducted on the same social media platform to align with the target audience. To recruit potential CAB members, the study team leveraged Twitter Ads Manager’s Promoted Ad feature to create and disseminate targeted advertisements aimed at parents with children aged 9‐14 years in the United States. At the time this study took place, the platform allowed extensive targeting capabilities and enabled the team to specify demographic filters and audience interests for purposive recruitment. Figure 1 shows an example of these recruitment ads.

In May 2020, parents were recruited using Twitter‘s “Promoted Ad” feature, which allowed the team to target broad user groups by specifying specific demographics, interests, and online behaviors and connections [24], making it a useful tool for online CAB recruitment as well as study participant recruitment more broadly. All advertisements were tailored based on age, gender, location, and language. To maximize outreach and engagement within the online environment, the study team optimized targeting by filtering for specific “keywords,” “interests,” “conversation topics,” and “follower look-alikes” to reach parents with children aged 9‐14 most likely to engage with the intervention platform. Table 1 provides a detailed breakdown of ad parameters, including follower look-alikes targeting, where the ads targeted parents with similar interests to followers of popular parent-related accounts on Twitter.

Interested parents were directed to an online screening survey linked within the promoted ads. The survey collected demographic information, Twitter and other social media usage patterns, and HPV vaccine awareness. Multimedia Appendix 1 provides the full list of screening questions. Parents who met the selection criteria (ie, used Twitter at least once a week, had a child aged 9‐14 years, and agreed to be contacted by the study team) were then invited to online interviews conducted through videoconferencing by a member of the study team.

The interview guide, provided in Multimedia Appendix 2, collected information about the individual’s background, interest in the project topic (ie, HPV vaccination), and assessed their fit as potential CAB members. The interviews also served as an opportunity for the study team to provide more detailed information about the project, the online nature of the CAB, member responsibilities, compensation, and next steps. CAB responsibilities described included the anticipated time commitment (ie, 10 online meetings over one year beginning in August 2020).

**Table 1.** Twitter ad parameters used for recruiting parents to the community advisory board in May 2020.

Parameter	Definition	Ads for mothers	Ads for fathers
Sex	—^a^	Female	Male
Age range (years)	—	25-54	25-54
Location	—	United States	United States
Keywords	Target or exclude people who searched for, tweeted with, or engaged with keywords.	Mom Parent Family Health Parenting	Dad Parent Health Parenting Family
Interests	Choose broad or narrow interests to reach the right people	Parenting teens Health news and general info	Parenting teens Health news and general info
Conversation topics	Target people who tweet about or engage with specific conversation topics.	Parenting teens	Parenting teens
Follower look-alikes	Target people with interests similar to an account’s followers. For example, enter @TwitterAds to target people likely to be interested in advertising on Twitter.	@ScaryMommy @AmerAcadPeds @MomCentral @RookieMoms	@FatherlyHQ @LifeofDadShow @AmerAcadPeds

a Not available.

### Engagement

CAB members collaborated with the study team to establish key activities, leadership structure, and operating principles. The group collectively determined meeting frequency, refined member roles and responsibilities, and reviewed the online research processes, including obligations to the University’s Institutional Review Board, as well as the study design and procedures. The remote nature of the CAB allowed for us to adopt a flattened hierarchy to ensure equal responsibility and input among members without a formal leadership structure.

Recognizing the importance of capturing meaningful, systematic feedback in an online setting, the study team developed interactive online workbooks that included questions, examples, and open-ended prompts to elicit parent input on project materials. These workbooks were shared digitally with CAB members one week before each scheduled monthly meeting, as members indicated this to be the optimal time frame for preparation. Completed workbooks were analyzed by the study team to identify themes for discussion during meetings. The workbooks served as a structured and accessible tool for gathering organized feedback on topics such as parenting experiences and HPV vaccine perceptions. Multimedia Appendix 3 provides an example workbook used to solicit input and feedback on developing non-narrative messages for the intervention.

For virtual meeting logistics, each session began with a short icebreaker to build rapport among members, workbooks were then discussed at length, giving CAB members an opportunity to provide real-time feedback on workbook topics. All meetings were recorded, and meeting minutes were distributed digitally to both the study team and CAB members, ensuring those unable to attend (which was rare) could stay updated. This online approach enabled flexibility and sustained engagement, accommodating busy schedules and parental responsibilities.

Feedback from the CAB was incorporated iteratively into the study. The study team maintained transparency through regular online updates, presenting how CAB suggestions influenced the intervention design and delivery. This iterative process strengthened trust and collaboration in a fully online setting.

### Maintenance

To evaluate the success and identify challenges encountered by CAB members during the first year, an anonymous midterm was conducted in July 2021, using an online survey and videoconference discussions at the end of the first year. The evaluation included measures examining the overall member satisfaction, meeting logistics, quality of activities and involvement, and what they envisioned for the future of the CAB. Multimedia Appendix 4 provides the full list of midterm evaluation questions.

**Figure 2.**
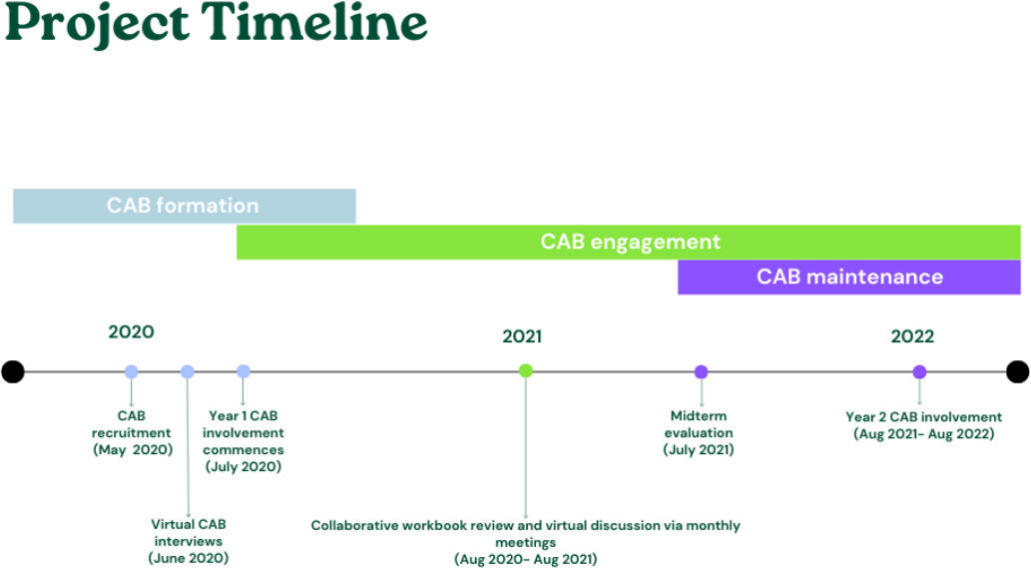
Project timeline of community advisory board activities (May 2020-August 2022). CAB: community advisory board.

To encourage sustained engagement, multiple digital strategies were used, including compensating members for their time and participation (ie, US $40 for workbook preparation and meeting attendance), conducting regular online meetings (ie, monthly during the first year) through videoconferencing, and sharing annual progress presentations to highlight study progress and CAB contributions to the study. To further celebrate their involvement, CAB members were provided a certificate of membership at the conclusion of the 1-year commitment. CAB members were invited to continue their participation for another year, extending their advisory role until August 2022. After completing the midterm evaluation, CAB activities were adjusted based on feedback collected and the remaining activities in the project lifecycle. Figure 2 provides an overview of the CAB activity timeline.

### Ethical Considerations

Evaluation activities were deemed exempt by the University of Arkansas Institutional Review Board (protocol 2108351667). All results presented in this manuscript are anonymous or deidentified, with no identification of individual CAB members included in the paper or supplementary materials. In addition, CAB members were compensated for their time and participation in the project, receiving US $40 in e-gift cards per meeting (US $20 for completing a workbook and US $20 for attending the meeting).

## Results

### Formation

In May 2020, as a part of a targeted social media recruitment campaign on Twitter, 17 parents completed the CAB screening survey. Of the 17, 6 responded to requests for an interview through videoconferencing. Following these interviews, all 6 parents were invited to join the parent advisory board, with all 6 accepting the offer. Table 2 details the CAB membership consisting of 5 mothers and 1 father, reflecting diversity across several key dimensions. The CAB represented diversity in terms of geography (Texas, Illinois, Pennsylvania, Indiana, Georgia, and Washington DC), community-type (urban, suburban, and rural), vaccination experience (half have vaccinated their child, though none were against getting the HPV vaccine for their child), and race and ethnicity (Black or African American, South Asian, Hispanic, and White).

Parents committed to 1-year involvement, which included participation in monthly online meetings. At the conclusion of the first year, 2 members stepped down due to personal commitments, while 4 members elected to remain active advisors to the project for an additional year, until the project’s completion.

**Table 2.** Community advisory board demographic composition (deidentified), July 2020.

Parental role	Race and ethnicity	Location	Community type	Vaccination perspective
Mother	Black	Washington, DC	Urban	Vaccine advocate
Mother	White	Indiana	Rural	Vaccine advocate
Mother	White	Georgia	Suburban	Vaccine hesitant
Mother	Hispanic	Pennsylvania	Urban	Vaccine advocate
Mother	South Asian	Maryland	Suburban	Vaccine advocate
Father	Black	Texas	Urban	Vaccine advocate

### Engagement

During the first year of the study, the CAB actively participated in 11 1-hour meetings conducted through Zoom (Zoom Communications). Meetings were held in the evening, typically 7 PM Eastern Time, to best accommodate parents’ schedules across time zones. As the study transitioned into its second year and moved into the implementation phase, meeting frequency was reduced to six 1-hour meetings over one year. This adjustment reflected the reduced need for iterative feedback.

The CAB provided valuable feedback on a range of HPV vaccine topics informed by their lived experiences as parents. Key contributions included identifying scientifically relevant messaging for the HPV vaccine, developing personas and refining the storylines used in the narrative communication intervention [25], co-creating Twitter messages, and reviewing study recruitment ads. Table 3 describes how CAB input informed various aspects of the intervention, as well as survey refinement and pilot testing of materials.

CAB involvement ultimately led to the development of a 2-week HPV vaccine intervention delivered on Twitter. The intervention included eight topics related to the HPV vaccine: (1) character introductions, (2) vaccine normative beliefs, (3) vaccine knowledge and awareness, (4) vaccine accessibility, (5) vaccine safety, (6) HPV vaccine for boys, (7) vaccine disparities and equity, and (8) cancer prevention. Multimedia Appendix 5 provides examples of the first two topics, or “chapters,” of the program, entitled “Meet the parents!” and “Parents are getting their kids the HPV vaccine.”

**Table 3.** Examples of community advisory board’s impact on quality, reach, and outcomes of the intervention (July 2020-August 2022).

Topic	Timeframe	CAB^a^ activity goal	CAB feedback and integration
CAB formation and project integration	July 2020-December 2021	Create CAB operating procedures Inform study activities with CAB input and feedback.	“Having the full study team present is not a problem-it is actually an advantage to be able to put names and faces together” “I really enjoy the breakout rooms and feel we can speak about the topic but also get to know the other members a bit better.” “I like the mix of face to face zoom meetings and pdf workbooks.”
Persona development	January 2021-May 2021	Alter personas to be more relevant and relatable.	“Many elements of this [persona] description remind me of myself or some in my closest circle.” “As I was reading [the persona descriptions], I could name specific people in my life who fit the characteristics of each character.”
Intervention content creation	April 2021-August 2021	Identify key themes for presenting information in the intervention. Review narrative intervention content.	“Knowledge and awareness has to come first to introduce the HPV vaccine into the general conversation. With information about accessibility, the audience can learn that [HPV is] not something that only certain people can get…” “Love the images and think they will be effective for communities of color in particular and all parents in general.” “The use of gifs and memes is great.”
Survey development	April 2021-December 2021	Ensure questions are relevant to parents and their vaccine decision making.	“People have very different relations with their providers and that plays a role in their decision making, making it important to include.”
Study recruitment	November 2021-August 2022	Develop recruitment ads with relevant language and images.	“I’d like more graphics to share for recruitment”
Intervention delivery	December 2021-August 2022	Advise on the various ways parents use Twitter. Advise on the logistics of implementing the intervention on Twitter.	“The order of tweets showing up in the feed is confusing and out of order.” “Using [Twitter] threads [to deliver the intervention], makes the most sense.”

a CAB: community advisory board.

### Maintenance

Table 4 shows the summarized themes from the midterm evaluation, and the full list of evaluation questions is provided in Multimedia Appendix 4.

**Table 4.** Qualitative themes of the anonymous community advisory board (CAB) midterm evaluation (July 2021).

Theme	Subtheme	Illustrative finding
Function of the CAB^a^	Frequency Convenience	Online meetings after normal business hours work best. Parents are happy with the meeting time and meeting frequency, and some would like to meet more often.
CAB research engagement	Confidence Knowledge building	All parents expressed confidence in describing the project and purpose of the advisory board.
		Everyone feels a great deal more knowledgeable about HPV^b^ and the HPV vaccine since joining the advisory board. All parents thought their input was well incorporated into the project.
Quality of the CAB	Comfort Collaboration	Parents largely feel safe and comfortable sharing ideas and opinions. Some discomfort sharing unpopular opinions (eg, vaccine hesitancy).
		Parents really enjoy working with one another and appreciate the connection they have with other board members.
Next steps for the CAB	Increased diversity	All parents feel that it would be beneficial to recruit additional, more diverse people to join the advisory board. Parents have an interest in participating in additional research activities

a CAB: community advisory board.

b HPV: human papilloma virus.

The midterm evaluation highlighted a high level of satisfaction among CAB members regarding their experience as advisory board members. Members particularly valued the connections they formed with other parents and their ability to contribute meaningfully to research in real time. All 6 CAB members reported that the monthly meeting frequency, timing, and format were well-suited to their needs as parents. However, 2 members suggested that additional meetings could further strengthen their connections with other members.

[Meeting logistics are] just right. It would be nice to maybe have a meet-up that isn’t HPV-related so we can bond more. I really enjoy getting to know everyone.

[CAB member 4]

I don’t know that anything can be done to improve the format. I think the team has done a very good job of facilitating the meetings.

[CAB member 1]

I love the communication! Everyone is so nice and fun to speak with. Even when we disagree it is never an issue. Maybe we should have a group chat.

[CAB member 5]

CAB members unanimously expressed confidence in describing the project to other parents and indicated that they gained more knowledge related to HPV and the HPV vaccine since joining the advisory board.

I personally felt like I was knowledgeable about HPV and the vaccine but this experience helps me understand it on a more deeper and informative level.

[CAB member 4]

The presentation we had regarding the HPV vaccine helped give me a clear understanding of the history, efficacy and uses for the vaccine. I have used some of the information that I learned and have used it while talking to friends and family about the HPV vaccine.

[CAB member 3]

I think I just took for granted that I knew enough about HPV and the HPV vaccine to understand it as being one more required shot kids need. I assumed it to be a simple STD, but now appreciate the wider repercussions of not getting vaccinated against it.

[CAB member 5]

The most enjoyed aspects or activities of the CAB members were expressed as: being part of a group that is improving the lives of their children, learning about HPV vaccination, learning about other parent perspectives, and seeing the project grow, being a part of a parent-led group, contributing to scientific research, and learning about HPV more broadly.

That I am actually a part of something that will help my son and generations to come.

[CAB member 5]

It has been really enjoyable to watch the project grow from the beginning to where it is now. I’ve also enjoyed hearing everyone’s different perspectives.

[CAB member 3]

Our group was able to create an authentic appreciation of the importance for work like this. It’s not just another social media survey, but real scientific research.

[CAB member 4]

Despite the positive feedback, areas of improvement were identified. While all 6 members expressed comfort with sharing their input with the full project team and CAB members, 5 felt more comfortable doing so during the smaller breakout sessions. This underscores the need to create a safe and inclusive space for parents to voice diverse or unpopular opinions, including vaccine hesitancy. In addition, members emphasized the importance of recruiting more parents with diverse backgrounds and perspectives to enrich group discussion, particularly those with differing views on vaccination.

I do think we need new members to diversify the group and add new voices and ideas.

[CAB member 3]

I feel that most everyone on the team is in complete support of the vaccination and is not really open to my questioning of the vaccinations.

[CAB member 2]

It always takes me a little while to warm up to new groups, but a tone of openness and support was established early on that made our group a safe space!

[CAB member 6]

## Discussion

### Principal Findings

This study describes strategies used for forming, engaging, and maintaining an online CAB for a parent-focused HPV vaccine intervention developed for social media. We strategically leveraged social media tools to recruit parents from the same social media platform where the intervention would be delivered (ie, Twitter). To engage with the CAB, we implemented interactive workbooks to ensure that every member had opportunities to provide input and feedback on intervention content and study direction. These digital tools streamlined the collection of data and facilitated meaningful discussions despite geographic barriers. An anonymous midterm evaluation was conducted to assess the first year’s outcomes and build upon successes, with 4 of the 6 CAB members continuing to advise on the project during its final second-year phase.

By using social media–based strategies to establish the CAB, we successfully increased the relevance of health materials for parents, improved the interventions reach, and enhanced its overall quality. The recommendations from the CAB significantly influenced the development and delivery of the intervention, while the online format enabled greater flexibility and accessibility for participants. To guide this process, we applied and adapted the Newman et al (2011) framework for community advisory boards, synthesizing best practices of community-based participatory research to focus on three key domains: formation, engagement, and maintenance. These domains provided an essential structure for successful operations, ensuring that the virtual CAB we formed effectively represented community interests and contributed effectively to the research process [2].

### Online CAB Formation

We found the need to clearly define the unique purpose, roles, functions, and responsibilities of an online advisory board to be paramount and supported by previous work [26]. There was great utility in applying Newman’s framework, which was originally designed for in-person CABs, to the development of online CAB [2]. While it can be challenging to build trust, transfer accurate knowledge, and maintain communication in in-person interactions, these challenges can be further amplified in the online environment as other researchers have also noted [19,27,28]. To address this, our approach emphasized establishing clear digital communication strategies, fostering a sense of community that grew beyond the project, and ensuring that all members felt equally involved and respected. Our study builds on these principles and details the promising approaches we encountered so that future research can replicate our processes of building a supportive environment and collaborative spirit, as online interventions and online advisory boards continue to expand.

Social media played a critical role in the recruitment and formation process of the CAB and this finding adds to the growing body of evidence supporting this important and effective tool [27]. By using Twitter’s targeted advertising tools, in addition to our recruitment survey and interviews, we reached a population of parents with diverse thoughts, backgrounds, and parental experiences. We prioritized the recruitment of vaccine naïve parents who were interested in learning about the HPV vaccine. Notably, intention to vaccinate their child was not a factor in determining who was interviewed or selected for membership, allowing us to include a wide range of perspectives. This decision draws from research demonstrating the need to be intentional in building an advisory board with diverse experiences and perspectives [29]. By embracing this diverse, flexible, and forward-thinking approach, we were able to create an online CAB that was resilient, adaptable, and invested in the project’s success.

Recruiting advisory board members in May 2020, at the onset of the COVID-19 pandemic, presented unique challenges to research also experienced in other studies [28]. While most of society was just beginning to adapt to changes in work and communication structures, this project had already committed to forming an online CAB to align with the online nature of the intervention. This early commitment to the online CAB and the parents’ intention to participate in a child-health focused research topic, likely contributed to strong engagement with the project tasks despite the quickly changing nature of the pandemic, especially in early 2020. In addition, the digital nature of the CAB and intervention allowed the project to move forward seamlessly during periods of mandatory lockdowns and rapidly changing guidance. This decision to prioritize an online framework also highlighted the usability and effectiveness of online CAB models in health research.

### Online CAB Engagement

Flexibility was vital for effective online CAB engagement. For parents, various factors such as work, holidays, long weekends, school events, personal obligations, or religious observations can affect their ability to respond timely, attend meetings, and complete tasks. By anticipating these factors, researchers can enhance participation and sustain engagement. To accommodate these needs, we used strategies such as asynchronous activities, including digital workbooks, to collect feedback systematically and reliably. This approach builds from the successes of other studies, demonstrating the effectiveness of flexibility and asynchronous activities [30]. These workbooks allowed CAB members to provide input at their convenience and served as a reliable tool for integrating their feedback into the intervention.

During online meetings, the use of videoconferencing tools facilitated dynamic discussions and collaborative decision-making. This approach reinforced expectations of ongoing participation and capitalized on the momentum of active collaboration. Meetings were recorded and made available to those who could not attend in real time (though this was rare), further enhancing flexibility and engagement.

Engaging parents through Twitter also provided an important avenue for buy-in and oversight of research activities. Parents’ familiarity with the platform facilitated a sense of ownership and connection to the intervention, which was ultimately delivered on the same social media platform. Their breadth of experiences and depth of understandings provided important grounding in the study’s health messaging element and enriched its development. This approach demonstrated the ability to use social media not only as a recruitment tool but also as a means for ongoing engagement with community stakeholders.

### Online CAB Maintenance

Maintaining and sustaining an online CAB required a strong emphasis on diversity, transparency, and active engagement to ensure the board’s long-term effectiveness and participation. The small membership size (n=6), allowed for personalized interactions and the cultivation of strong connections among members. “Icebreakers” and informal conversations during meetings were particularly effective for building camaraderie, uncovering common interests, and strengthening the sense of community within the CAB, an approach that has demonstrated effectiveness across studies and populations [2,26,28].

Frequent meetings were instrumental in explaining project activities and tasks, the purpose tasks (ie, workbooks), and demonstrating how CAB contributions were integrated into the intervention. This “give-back” approach, fostered a strong sense of ownership and commitment among members, as they could see the tangible impact of their participation. To sustain engagement between meetings, members were encouraged to stay connected through online platforms, such as LinkedIn, or private group cats, creating additional opportunities for collaboration and relationship building.

The digital-first format also allowed us to celebrate member contributions in meaningful ways. For example, certificates of membership were shared electronically, and members received regular updates on how their feedback had influenced the intervention’s development. These findings, demonstrating the effectiveness of real-time updates, adds to existing evidence to support the approach [19,26]. These practices emphasized the value of their input and strengthened their motivation to continue participating with the project.

### Limitations

Our approach had several limitations worth noting. First, we began recruiting CAB members during the height of the COVID-19 pandemic, a period marked by widespread skepticism of online information and heightened vaccine-related distress. These factors may have limited the number of people interested in the project, particularly for those hesitant about engaging in a vaccine-related initiative. Furthermore, as the pandemic continued, fatigue and continued experienced by parents and the broader public may have contributed to a slight drop-off in CAB membership between years 1 and 2.

Second, while social media platforms provided effective tools for recruitment, reliance on Twitter alone may have excluded individuals or limited diversity. Expanding recruitment efforts to include additional platforms could diversify participant pools and enhance inclusivity in future studies. Furthermore, increasing the diversity of the CAB, whether in terms of age, race, gender, disability status or viewpoints, would further enrich discussions, better represent the community, and ultimately better inform the research. For instance, incorporating parents who are not digitally native or who face barriers to social media engagement could offer valuable perspectives often overlooked in online-only recruitment strategies.

We also recognized the underrepresentation of fathers on the CAB (n=1). Although Twitter’s targeting tools allowed for gender-based recruitment, leveraging specific social media groups or communities tailored to fathers, such as online parenting forms or father-focused groups, may help address this imbalance. Leveraging connections to recruit more fathers could be a valuable strategy for future studies that seek to engage parents, as increased representation and diversity only broadens the range of perspectives and ensures the CAB truly reflects the community it serves. Importantly, while underrepresentation of fathers may be viewed as a limitation, our formative research found that fathers were more likely to defer to the mother regarding child health decisions, perhaps explaining their underrepresentation in this study.

While the online format offers flexibility and accessibility, some CAB members noted challenges related to ensuring that all voices, particularly those with differing opinions, were fully incorporated into the project. Online group discussions may sometimes discourage dissenting opinions due to the absence of in-person cues or the fast-paced nature of digital conversations. Using structured feedback mechanisms, such as anonymous surveys or one-on-one digital interviews, may help future online CABs better capture a broad range of perspectives and ensure that all voices are equally heard. The online model reflected how the study was developed and was essential for navigating the constraints of the pandemic; however, it inherently posed limitations for fostering the depth of connection and collaboration that may arise in face-to-face interactions. Future studies could consider incorporating hybrid models, blending online meetings with occasional in-person events, to balance flexibility of online formats with the relational benefits of in-person collaboration.

Despite these limitations, we believe that an online CAB offers unique opportunities to engage diverse populations, facilitate inclusive participation, and provide meaningful contributions to health programs delivered online. By iterating on these approaches and addressing the highlighted limitations, future research can further refine online CAB methodologies and amplify their impact in public health initiatives.

### Conclusion

This study demonstrates the feasibility and effectiveness of using online and social media–based CABs in health research. Textbox 1 shows a list of guiding principles for developing online CABs based on findings from this study and supported by previous research [26-30]. Future research should explore additional methods for integrating digital tools to further enhance engagement and maintain long-term participation in online CABs.

Online and digital health interventions have become influential tools for public health, providing innovative means to address health disparities and influence health behaviors on a broad scale. As online interventions continue to expand, so must our efforts to strategically and effectively engage online advisors within the community. Social media and online platforms serve as powerful avenues for recruiting diverse and representative CAB members, fostering active engagement in research processes, and generating quality feedback that can significantly enhance research programs. These platforms not only enable the inclusion of voices from geographically dispersed and underserved populations, but also create opportunities for dynamic, real-time collaboration that aligns with the evolving digital landscape. By leveraging these approaches, researchers can maximize the potential of online CABs as flexible, scalable, and inclusive mechanisms for participatory research. This model holds promise for driving meaningful health outcomes, reducing inequities, and fostering a deeper connection between research initiatives and the communities they aim to serve.

Textbox 1. **Guiding principles for developing online community advisory boards.**

Forming the online community advisory board:

- Clearly define the purpose, roles, functions, and responsibilities expected of the online community advisory board members, ensuring responsibilities align with the format and digital engagement.
- Leverage social media platforms and tools, such as targeted ads and other adjustable parameters to recruit diverse members from specific demographics.
- Prioritize diversity in geography, online behaviors, and parental perspectives by using digital methods to reach underrepresented groups and those with varied social media experience.

Engaging the online community advisory board:

- Use digital tools like interactive workbooks and online surveys to collect feedback systematically, ensuring ease of access and usability in an online environment.
- Build a sense of community and connection by facilitating collaborative discussions during video calls and leverage shared online spaces to connect members meaningfully, as whole people, despite geographic distance.
- Ensure flexibility in scheduling and participation, leveraging asynchronous tools such as recorded meetings, digital materials, and follow-up communication to accommodate members’ busy online and offline schedules.

Maintaining the online community advisory board:

- Enhance inclusivity by using social media and other online platforms to recruit members from diverse online communities, ensuring broad representation across digital spaces and perspectives.
- Foster a sense of belonging by incorporating icebreakers and encouraging casual online interactions, such as LinkedIn connections or private text or chat groups to sustain engagement between meetings and outside of the project.
- Continuously demonstrate the impact of members’ input by providing real-time updates through online platforms and integrating feedback into the digital intervention to maintain motivation and accountability.

## Supplementary material

10.2196/65986 Multimedia Appendix 1 Screening questions for potential community advisory board members, May 2020.

10.2196/65986 Multimedia Appendix 2 Community advisory board interview guide.

10.2196/65986 Multimedia Appendix 3 Sample workbook for community advisory board (CAB) members.

10.2196/65986 Multimedia Appendix 4 Parent advisory board year 1 midterm evaluation.

10.2196/65986 Multimedia Appendix 5 Human papillomavirus (HPV) vaccine content for Twitter co-created by community advisory board (CAB) members and study team.

## Abbreviations

CAB: community advisory board
HPV: human papillomavirus
