# Supplementary material for: Developing an Online Community Advisory Board (CAB) of Parents From Social Media to Co-Design an Human Papillomavirus Vaccine Intervention: Participatory Research Study
Source: JMIR Form Res. 2025 Apr 16;9:e65986. doi: 10.2196/65986 (PMC12017609; doi:10.2196/65986)
Supplement: Multimedia Appendix 1 [file formative-v9-e65986-s001.docx]

**Appendix 1. Screening Questions for Potential Community Advisory Board Members, May 2020.**

Thank you for your interest in becoming a parent advisor! Our project is about the HPV vaccine and how it protects against types of HPV infections that can cause cancer. For more information, please visit https://hashtaghpv.com/getinvolved/ Parent advisors will be compensated $40/meeting. Following brief survey, if you are eligible, someone from our project team will be in touch with you by email. Filling out this survey does not guarantee acceptance to be a parent advisor. Thank you for your interest!

**1) How did you hear about being a parent advisor on the project?**

Colleague/coworker | Friend of family member | Twitter | Facebook | Other

**2) How often do you go on Twitter?**

Several times a day | Once a day | Several times a week | Once a week | Less than once a week | Never

**3) Do you have at least one child who is between the ages of 9 and 14?**

Yes | No

**4) Does your child/children between the ages of 9 and 14 have any disabilities?**

Yes | No | Prefer not to answer

**5) Do you work for pay in any health-related position such as a doctor, nurse, hospital administrator, or receptionist in a doctor’s office?**

Yes | No

**6) Are you Hispanic, Latinx, or Spanish origin?**

Yes | No

**7) How would you describe yourself? Please mark all that apply.**

American Indian or Alaska Native | Asian | Black or African American | Native Hawaiian or Other Pacific Islander | White | Other

**8) We are looking for a mix of parents to serve as advisors. Please indicate your gender identity.**

Female | Male | Transgender | Non-binary | Other

**9) What year were you born? Slide the toggle to select your answer.**

Toggle 1950 - 2000

**10) Have you ever heard of the HPV vaccine?**

Yes | No

**11) Do you have any children who have started or received the HPV vaccine?**

Yes | No | I don’t know

**12) Do you agree to be contacted by a member of the project team?**

Yes, I agree | No, I do not agree

**13) Please provide your name for us to follow up.**

**14) Please provide your email address for us to follow up.**

**15) What is your Twitter username?**

**16) Please share any comments or questions you may have below.**
