# Supplementary material for: Developing an Online Community Advisory Board (CAB) of Parents From Social Media to Co-Design an Human Papillomavirus Vaccine Intervention: Participatory Research Study
Source: JMIR Form Res. 2025 Apr 16;9:e65986. doi: 10.2196/65986 (PMC12017609; doi:10.2196/65986)
Supplement: Multimedia Appendix 2 [file formative-v9-e65986-s002.docx]

**Appendix 2.** **Community Advisory Board Interview Guide**

| **Interview Guide Part One: Background Information** |
| --- |
| 1. To start, can you tell me a little about yourself?    1. Probes:       1. How have you been spending your time while in quarantine?       2. Have you participated in a CAB or research project before?       3. *If they indicate they have children at home:* How many children do you have at home and how have they been keeping busy? |
| 1. Now that I've learned a bit about you, can you tell me more about your interest in the CAB and #HPV project?    1. Probes:       1. What are your expectations of the CAB? |
| 1. Our research will mostly be based on Twitter. Can you tell me about your Twitter use?    1. Probes:       1. How often do you use the app?       2. Do you usually use it on your phone or on a laptop/tablet/desktop?       3. Do you ever use the app to get health information? |
| 1. Have you worked with other parents at your child’s school or in your community?    1. Probe:       1. Get more information about the capacity in which they worked with parents       2. More information about collaboration – did they give advice/input or listen to advice/input to fulfill a common goal? |
| 1. What experiences have you had related to your child’s health or preteen health? |
| 1. As a CAB member, you’ll be working with a mix of parents, researchers, and social media experts. Can you tell me about any experience you have working with people from other fields or backgrounds? |
| **Interview Guide Part Two: Administrative Information** |
| 1. The CAB will be completely virtual, and meetings will typically take place online, most likely using video or phone conferencing. Having access to a computer or mobile device during these meetings will likely be important to participate. Are you able to participate in virtual meetings?    1. Probes:       1. Are there any barriers/challenges to getting access to internet?       2. If yes, opportunity to say that they could also call into Zoom meetings if internet is difficult to access. |
| 1. We are asking CAB members to participate in about 10 meetings over the next year, and they will be conducted at various times, mostly once a month meetings but sometimes more or less often depending on where we are in the project. Would you be available to participate in 10 meetings as a CAB member?    1. Probes:       1. What time of day would be best for you to participate in meetings?       2. Are there any barriers/challenges to participating in meetings that need to be addressed? |
