# Supplementary material for: Developing an Online Community Advisory Board (CAB) of Parents From Social Media to Co-Design an Human Papillomavirus Vaccine Intervention: Participatory Research Study
Source: JMIR Form Res. 2025 Apr 16;9:e65986. doi: 10.2196/65986 (PMC12017609; doi:10.2196/65986)
Supplement: Multimedia Appendix 3 [file formative-v9-e65986-s003.pdf]

Recap

In the last meeting, we focused on the development of the personas. As you know, this project will consist of two groups (story and non-story). Parents in the story group will read Twitter posts that talk about the HPV vaccine through stories based on the characters that we are developing. The information about the HPV vaccine will be told through their stories and their experiences. We want to give "life" to the information and decisions that parents make about the vaccine.

The non-story group will see posts that focus mostly on evidence, such as numbers and facts, and do not include the character's stories and experiences. **For this workbook and meeting, we will focus on the non-narrative messages**.

On the next page, you will find 7 topics that we plan to use to guide our creation of the non-story messages. After we get your feedback on topics, we would also like to gather your thoughts on the messages and the sources that we used to create the messages.

Topics

Below are the 7 topic areas that we have chosen to represent through the non-story messages. On the pages following this, you will find sample messages and images that we plan to use for the non-story tweets. We will ask you for feedback on both the topics and the messages.

1. HPV Knowledge & Awareness

- What is HPV, age for vaccination, how do you know if your child has HPV, treatment for HPV, etc.

2. HPV Vaccine is for Cancer Prevention

- Facts and statistics on HPV and cancer.

3. HPV Vaccine Safety

- Information on the safety and effectiveness of the HPV vaccine.

4. HPV Vaccine Normative Beliefs

- Information on the high rates of vaccine acceptance among parents, number of children vaccinated, and beliefs that support HPV vaccination, etc.

5. HPV Vaccine Accessibility

- Recommended dosage, where to get the vaccine, etc.

6. HPV Vaccine For Boys

- Information specific to the HPV vaccine for boys.

7. HPV Disparities & Equity

- Vaccine coverage across communities, immunocompromised children, etc.

Feedback (Topics)

Rank the 7 topic areas in order of importance to you.

1.

2.

3.

4.

5.

6.

7.

Please explain why you chose your top 2 topics.

Please explain why you chose your bottom 2 topics.

Are there any other topic areas that you think would be important to include that we did not include already? Why or why not?

Do you think that any of the 7 topic areas that we chose would <u>not</u> be important to include? Why or why not?

Sample Messages and Images

HPV Knowledge & Awareness

Message	Image
At any given time, about 1 in 4 adults in the US have HPV — and it can lead to serious health problems. Find out how to help #EndHPVCancers with info from @HHSvaccines: https://bit.ly/2X15s8n	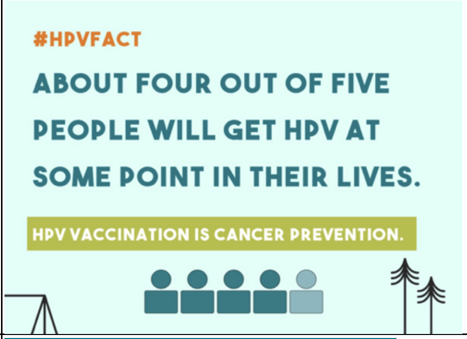
It's scary but true, most people infected with HPV don't know they have it. The good thing is we can protect children from HPV with two doses of a vaccine. #vaccineswork #vaccination #HPVvaccine #2Shots2StopCancer	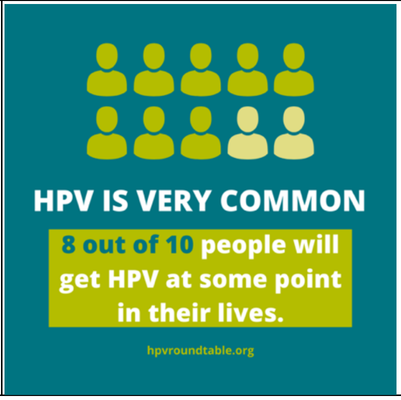

Feedback

What do you think of the sample messages/images for this topic area? Is there anything missing from the messages/images?

What do you think of the wording of the messages?

Do you think that this message communicates the same things that you would want to communicate about this topic to another parent? Why or why not?

HPV Vaccine is for Cancer Prevention

Message	Image
HPV vaccination for boys and girls at age 11 or 12 can help prevent #CervicalCancer. Get more info from @HHSvaccines to help #EndHPVCancers: https://bit.ly/2X15s8n	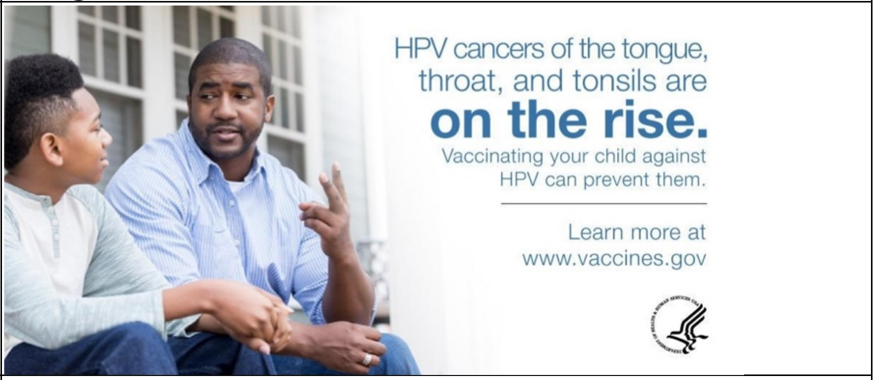
The HPV vaccine doesn't only prevent cervical cancer — it also helps protect against cancers of the tongue, throat, and tonsils.	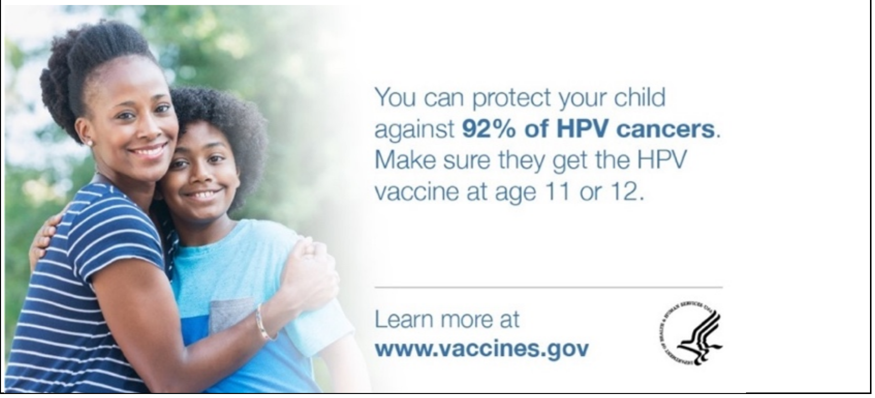

Feedback

What do you think of the sample messages/images for this topic area? Is there anything missing from the messages/images?

What do you think of the wording of the messages?

Do you think that this message communicates the same things that you would want to communicate about this topic to another parent? Why or why not?

HPV Vaccine Safety

Message	Image
The HPV vaccine has a strong record of safety — and it's an effective way to protect your loved ones against HPV cancers. Learn more from @HHSvaccines: https://bit.ly/2X15s8n	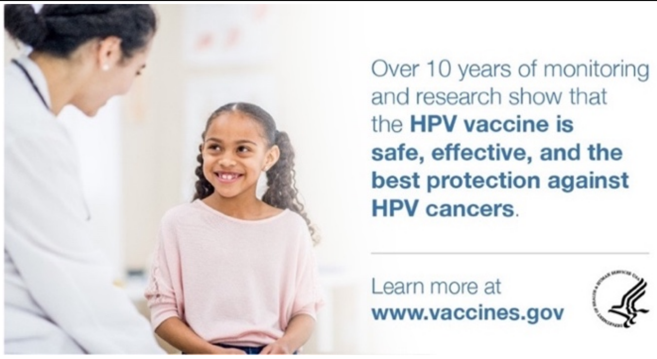
Like all vaccines, scientists monitor the HPV vaccine over time to ensure it's safe and effective. You can protect against 90% of HPV cancers by getting vaccinated. https://bit.ly/2X15s8n	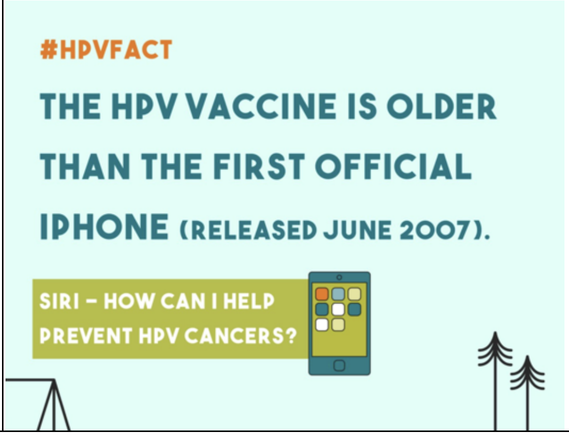

Feedback

What do you think of the sample messages/images for this topic area? Is there anything missing from the messages/images?

What do you think of the wording of the messages?

Do you think that this message communicates the same things that you would want to communicate about this topic to another parent? Why or why not?

HPV Vaccine Normative Beliefs

<table>
<tr><th>Message</th><th>Image</th></tr>
<tr><td>Most parents are choosing to get the first dose of the HPV vaccine to protect their children from cancers caused by HPV infections. Are your kids protected?</td><td>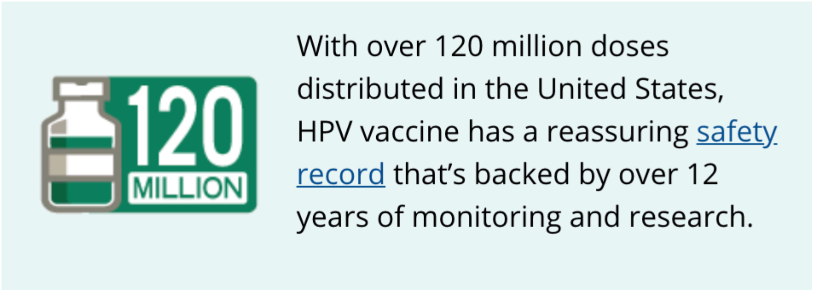
</td></tr>
<tr><td>Is your child 11 or 12? Talk to you child's doctor about getting the #HPV vaccine at their #backtoschool wellness visit. Learn more about the HPV vaccine and why it's important → http://bit.ly/2FBh4H7 #NIAM18</td><td>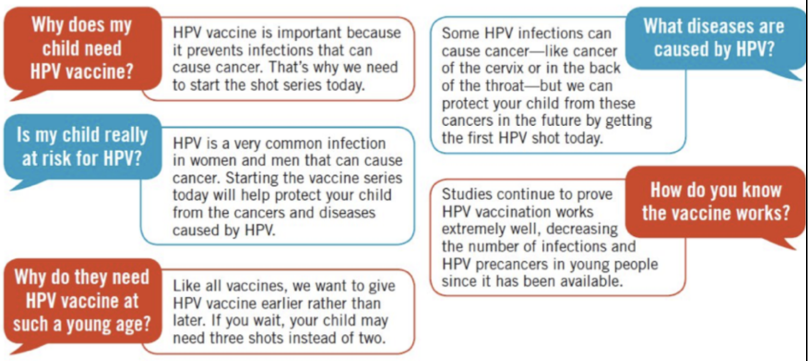
</td></tr>
</table>

Feedback

What do you think of the sample messages/images for this topic area? Is there anything missing from the messages/images?

What do you think of the wording of the messages?

Do you think that this message communicates the same things that you would want to communicate about this topic to another parent? Why or why not?

HPV Vaccine Accessibility

Message	Image
Protect yourself from HPV by getting an HPV vaccination. To find HPV vaccines in your area, use the Office of Population Affairs' Title X Family Planning Clinic Locator: https://opa-fpclinicdb.hhs.gov #CervicalCancerAwarenessMonth	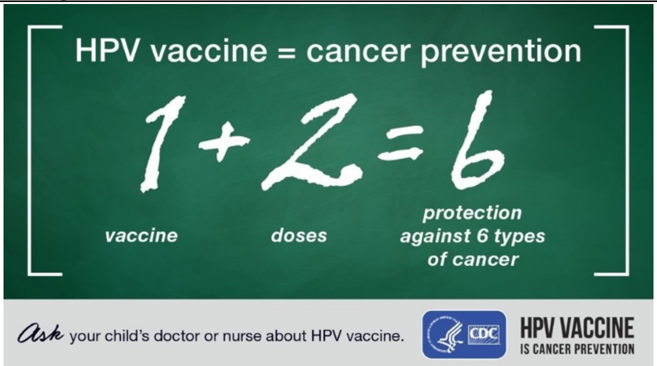
CDC recommends that boys and girls get vaccinated for HPV between 11 and 12 years of age. You can take advantage of any visit to your child's doctor to get recommended #vaccines for your child, including sports physicals or annual checkups. http://bit.ly/2iupQ1C #FAQFriday	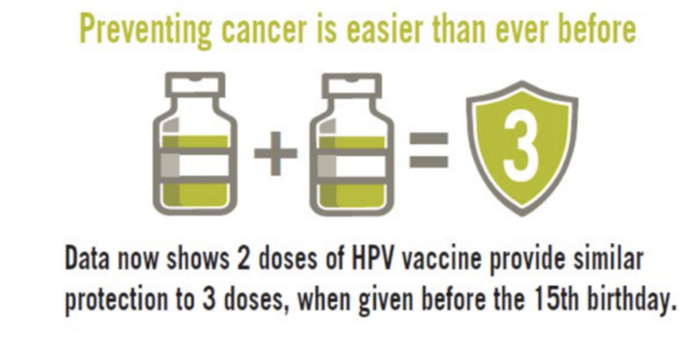

Feedback

What do you think of the sample messages/images for this topic area? Is there anything missing from the messages/images?

What do you think of the wording of the messages?

Do you think that this message communicates the same things that you would want to communicate about this topic to another parent? Why or why not?

HPV Vaccine for Boys

Message	Image
Did you know men are 4 times more likely than women to get HPV cancers of the tongue, throat, and tonsils.	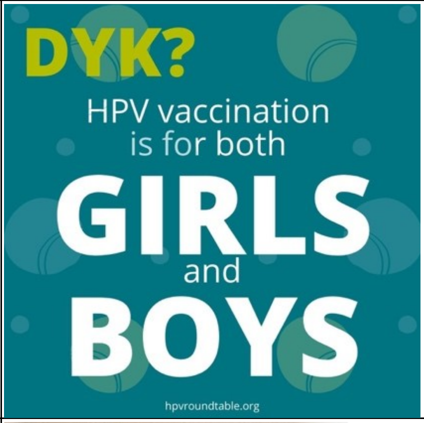
Don't wait to vaccinate! Doctors say it's #Time2Vax boys and girls between the ages of 9 and 12 with the HPV vaccine when #cancer prevention is most effective.	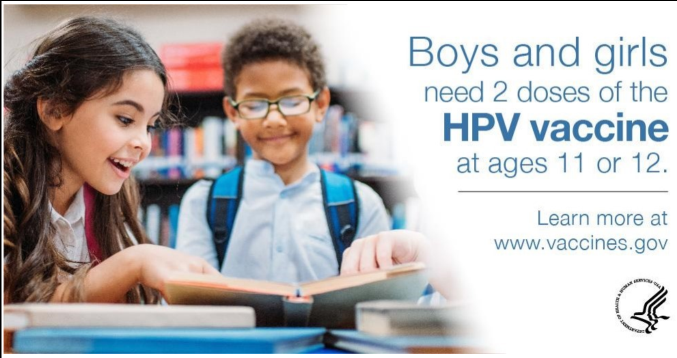

Feedback

What do you think of the sample messages/images for this topic area? Is there anything missing from the messages/images?

What do you think of the wording of the messages?

Do you think that this message communicates the same things that you would want to communicate about this topic to another parent? Why or why not?

HPV Disparities & Equity

<table>
<tr><th>Message</th><th>Image</th></tr>
<tr><td>In the U.S., it is estimated that over 13,240 new #CervicalCancer cases will be diagnosed in 2018. Despite widespread availability of pap testing, disparities in cervical cancer incidence and mortality rates by race persist. Black women have the highest mortality rates of the disease. #HPVvaccine can help #EndHPVCancer #2Shots2StopCancer</td><td>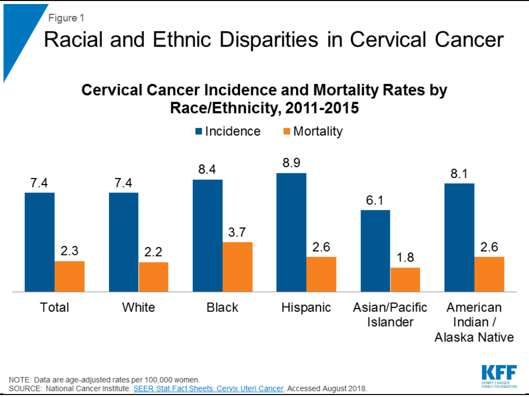
</td></tr>
<tr><td>Did you know? Adolescents whose families live below the poverty line and those living in urban areas were more likely to be up to date on their HPV vaccine compared to adolescents whose families live above the poverty line or live in rural areas. Let's #EndHPVCancer by signing up your child for the #HPVvaccine today.</td><td>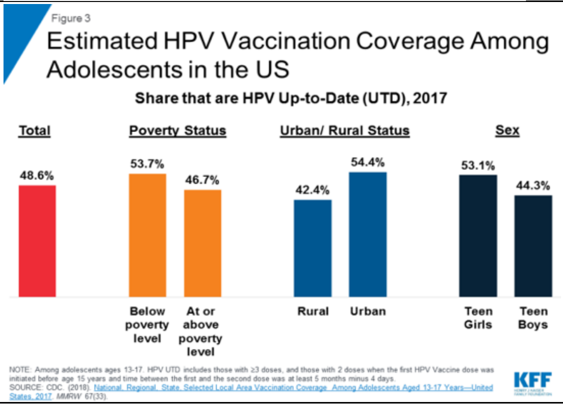
</td></tr>
</table>

Feedback

What do you think of the sample messages/images for this topic area? Is there anything missing from the messages/images?

What do you think of the wording of the messages?

Do you think that this message communicates the same things that you would want to communicate about this topic to another parent? Why or why not?

Sources

Below are the sources that we used to gather the messages and images:

Vaccines.gov @HHSGov
https://twitter.com/HHSGov

Centers for Disease Control and prevention (CDC) @CDCgov
https://twitter.com/CDCgov

American Academy of Pediatrics (AAP) @AmerAcadPeds
https://twitter.com/AmerAcadPeds

HPV Roundtable @HPVRoundtable
https://twitter.com/HPVRoundtable

Do you trust the sources that we used to find messages and images? Why or why not?

Tell us about any sources that you trust (other than your child's primary care provider) and use to find health/vaccine information (news sources, websites, organizations, social media accounts, etc.).

Overall Feedback

What questions do you have, or did you have about the HPV vaccine? Were any of the questions that you have about the HPV vaccine answered in any of these messages?

How might you respond to messages like these if you saw them on Twitter (ex. scroll past, like, retweet, comment, send to others, etc.)?

How much, if any, of the information in the messages and images was new to you?

If you were a parent who is still considering the HPV vaccine, would you find the information provided in these messages helpful? Why or why not?

Please share any other thoughts or feedback.

We appreciate your time and feedback! When you are finished with the workbook, please save and send back to Meredith Rose at mr3547@drexel.edu. We look forward to discussing your thoughts in the meeting!
