# Supplementary material for: Developing an Online Community Advisory Board (CAB) of Parents From Social Media to Co-Design an Human Papillomavirus Vaccine Intervention: Participatory Research Study
Source: JMIR Form Res. 2025 Apr 16;9:e65986. doi: 10.2196/65986 (PMC12017609; doi:10.2196/65986)
Supplement: Multimedia Appendix 4 [file formative-v9-e65986-s004.docx]

**Appendix 4.** Parent Advisory Board Year 1 Mid-Term Evaluation

1. What are your thoughts on the timing of the meetings? If a different time was offered, what would typically work best for you?
2. What do you think of the spacing of the meetings? (i.e. too far apart, too close together, or just right?)
3. What do you think of the virtual format of the meetings? Is there anything we could do to improve them?
4. How did you feel about having the whole research team (sometimes upwards of 8 people) at each meeting? Is there another format you would have preferred?
5. How much do you feel your input was used in research tasks?
6. Please share how we could have incorporated your input more in the research tasks.
7. Please share an example of how your feedback was incorporated into study activities and/or materials.
8. How would you describe the communication between the PAB and research team? What worked well and what can be improved?
9. How did you feel sharing your input with the larger group during meetings?
10. How did you feel sharing your input during the small groups (breakout rooms) during meetings?
11. What, if anything, did you like best about being part of the PAB?
12. What, if anything, did you not especially like about being part of the PAB?
13. How has your participation in the PAB affected your understanding and knowledge of HPV and the HPV vaccine?
14. Now being in the PAB for almost a year, how comfortable would you feel explaining the project and its goals to other people?
15. What are your thoughts on how we have addressed parental concerns? Are there any additional content areas or resources you were hoping to receive from us?
16. Thinking back to almost a year ago, you saw an ad for the PAB on Twitter, then filled out a survey. We first met by phone, and you were successfully selected for the PAB! What did you think of the recruitment process for the PAB? How can we improve the recruitment process for future studies?
17. How do you feel about the amount of information you were given in the lead up to completing the workbooks?
18. You generally were given 2 weeks to fill out the workbook. What are your thoughts on this turnaround time? (I.e. was this enough time, too much, or too little?)
19. What are your thoughts on the length of the workbooks?
20. The workbooks were done in a fillable PDF format. Do you have suggestions for another format we could use for future workbooks?
21. What are your thoughts on the clarity of the questions in the workbook? What are some ways we could improve the clarity?
22. What are some other ways we could further include you in the study?
23. What are some other formats we could use to collect your feedback?
24. We are considering increasing the number of PAB members. As a charter member of the PAB, and given your involvement over the past year, what are your thoughts on having new members? If you are interested in having new members, would you be interested in recruiting more parents from your networks?
25. We know participating in the PAB takes time, and as a parent your time is valuable! We want to make sure we are acknowledging the time you spend on the work, specifically in how we compensate you for your time. Do you feel you were properly compensated for your work? Are there other ways we could compensate you for your work?
26. As a PAB member, you have helped us refine our research methods and processes. We will be collecting data over the next year, and are considering having PAB members play a larger role in the research tasks. This could include (but is not limited to) helping us interpret data. Would you be interested in taking on more research tasks?
27. What are your thoughts on the diversity of the PAB? What are some ways we can improve the diversity of the group?
28. How likely would you recommend a fellow parent to join a PAB like this one?
29. Are there any other thoughts you’d like to share?
