# Supplementary material for: Developing an Online Community Advisory Board (CAB) of Parents From Social Media to Co-Design an Human Papillomavirus Vaccine Intervention: Participatory Research Study
Source: JMIR Form Res. 2025 Apr 16;9:e65986. doi: 10.2196/65986 (PMC12017609; doi:10.2196/65986)
Supplement: Multimedia Appendix 5 [file formative-v9-e65986-s005.pdf]

Project Hashtag HPV

About Team Resources Publications and Presentations Contact Join The Study

Narrative Content:

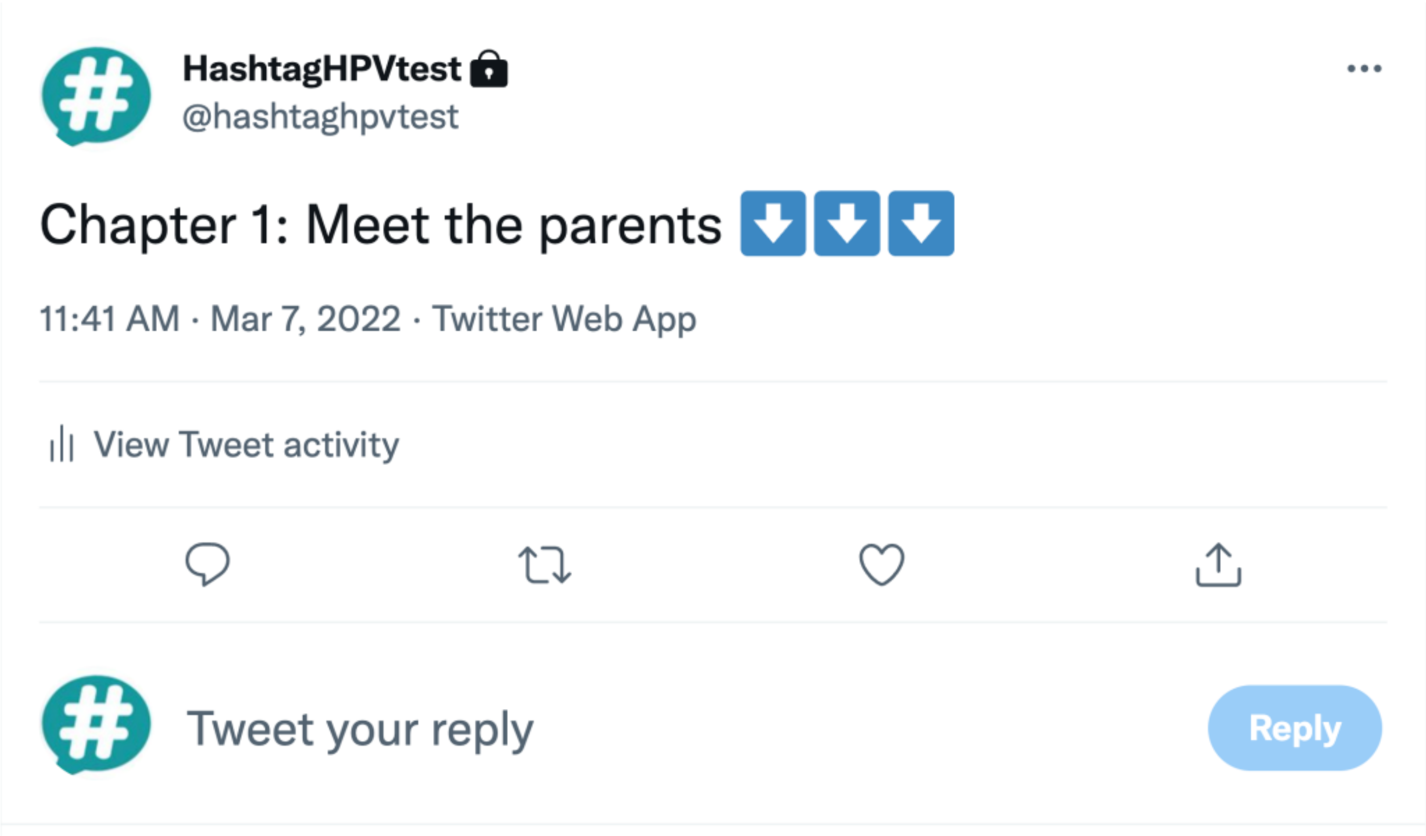

HashtagHPVtest @hashtaghpvtest · 21s

Replying to @hashtaghpvtest

The topic is the HPV vaccine. Four parents will discuss it. They started talking at school pick-up and have stayed in touch ever since. Turns out, their kids (and in one case, grandkids) visit the same pediatrician, Dr. Singh. Let's listen in. 1/6

1

HashtagHPVtest @hashtaghpvtest · 20s

While the parents in these stories aren't real, their experiences are. 2/6

HashtagHPVtest @hashtaghpvtest · Feb 15

👩🏻‍⚕️ Lin🩺: Hi, I'm Lin. I'm a staff nurse supervisor at a large local hospital. I've been married for 17 years. 2 kids: both tweens. I am very active in my community and recently became president of my neighborhood association. 3/6

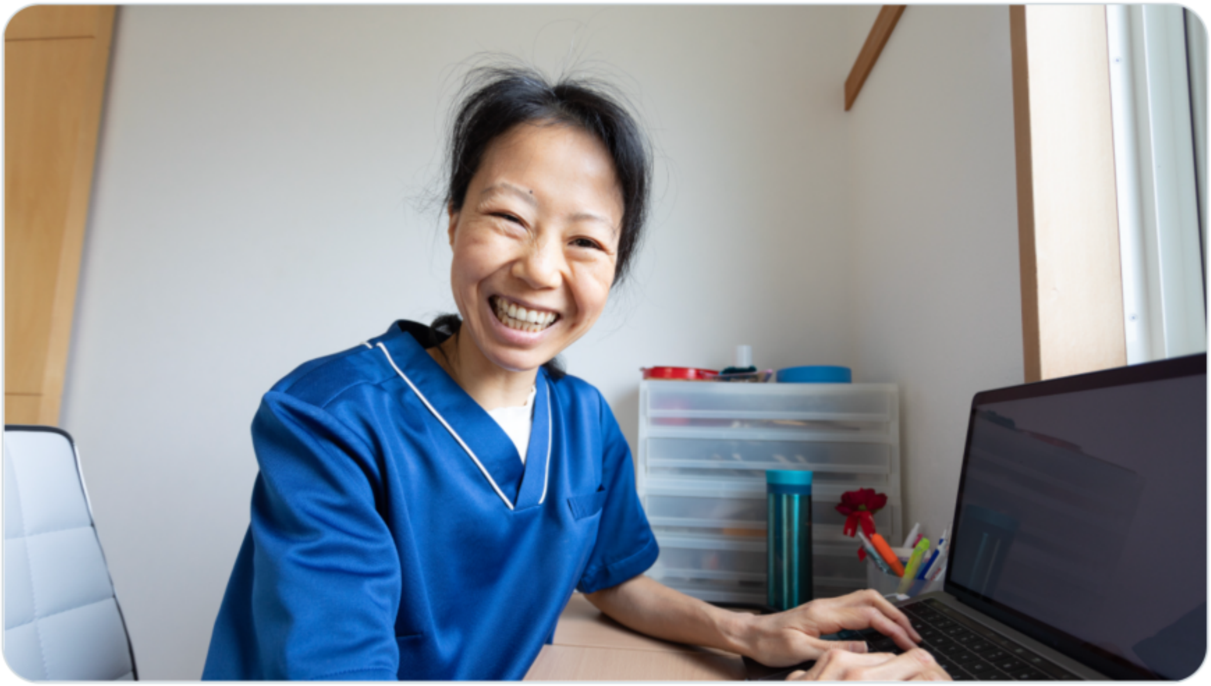

1

HashtagHPVtest 🔒 @hashtaghpvtest · Feb 15

👩🏾Dana☮️: Hi I'm Dana. Taking on the world with my 11 y/o while working two jobs, one at my local market & another driving with a rideshare company. Passionate about family, community, & social justice. Ask me anything. My friends will tell you I always have the answer. Lol! 4/6

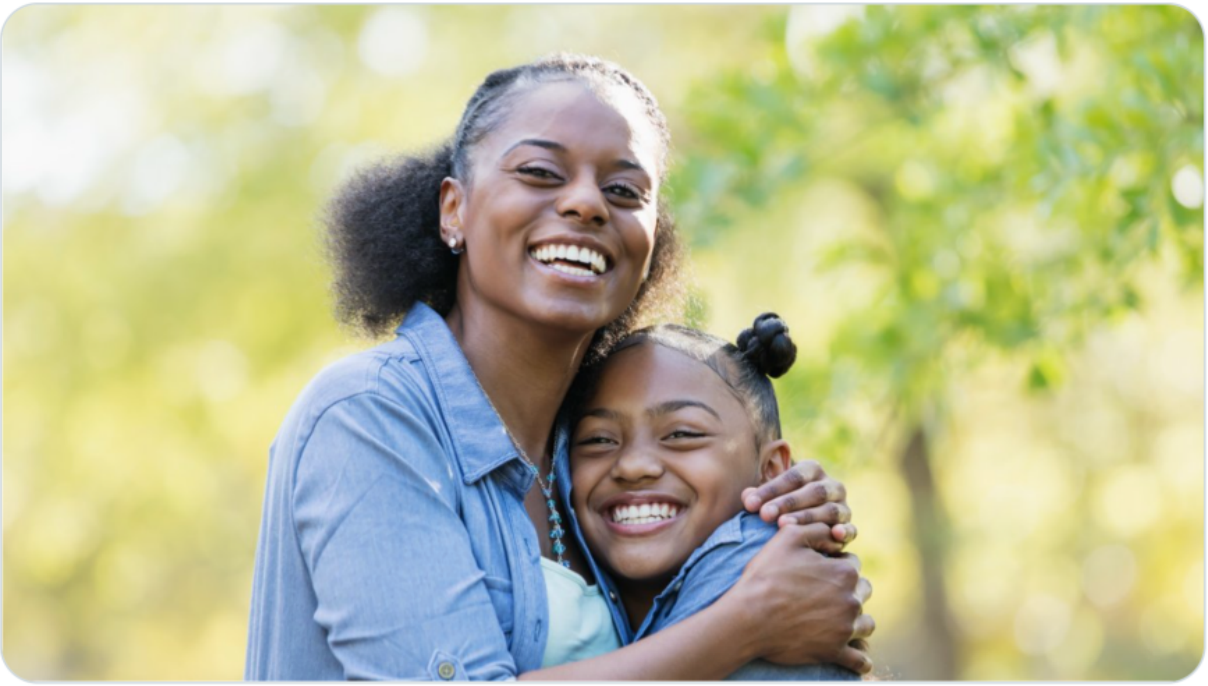

1

HashtagHPVtest 🔒 @hashtaghpvtest · Feb 15

👩‍💻Jessica⚽: I'm Jessica, dedicated mother of 3, soccer mom, at-home gymnastics coach, expert Googler, and avid blogger. Check out my blog for healthy recipes, organization hacks, and need-to-know info. 5/6

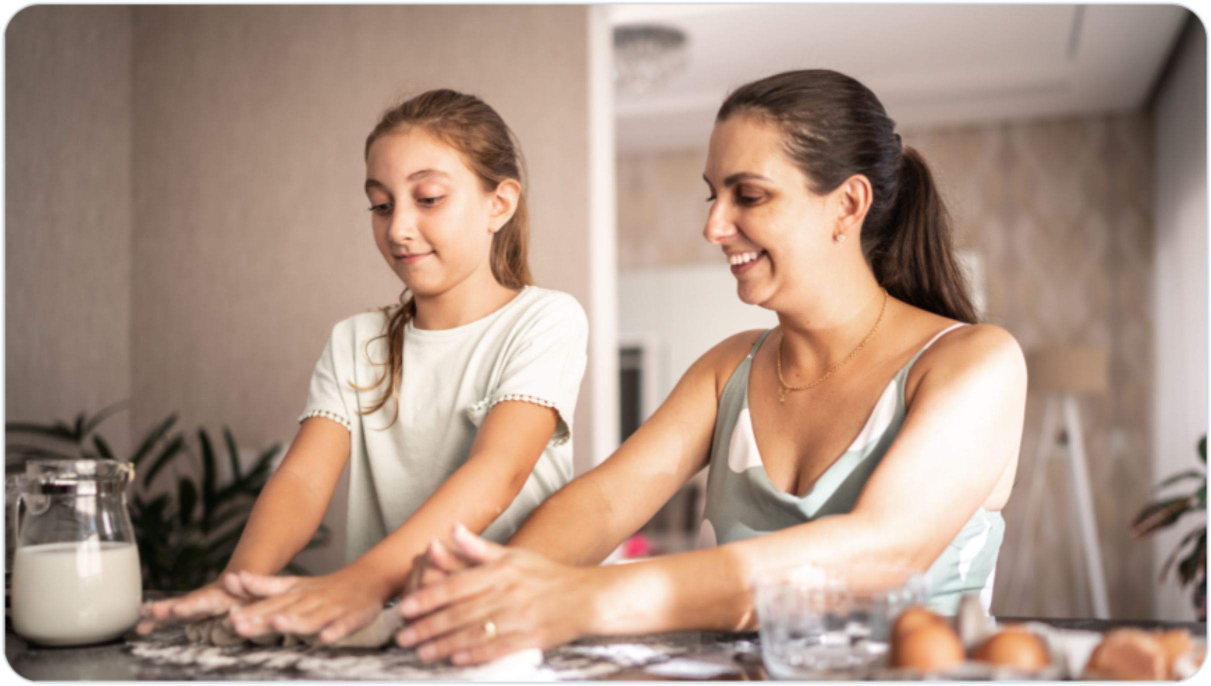

1

HashtagHPVtest 🔒 @hashtaghpvtest · Feb 15

👴🏽Miguel🛻: ¡Hola! I'm Miguel, retired high school counselor. Helping to raise 5 grandchildren is keeping me busier than ever. So is spending a lot of time helping my community through my church. 6/6

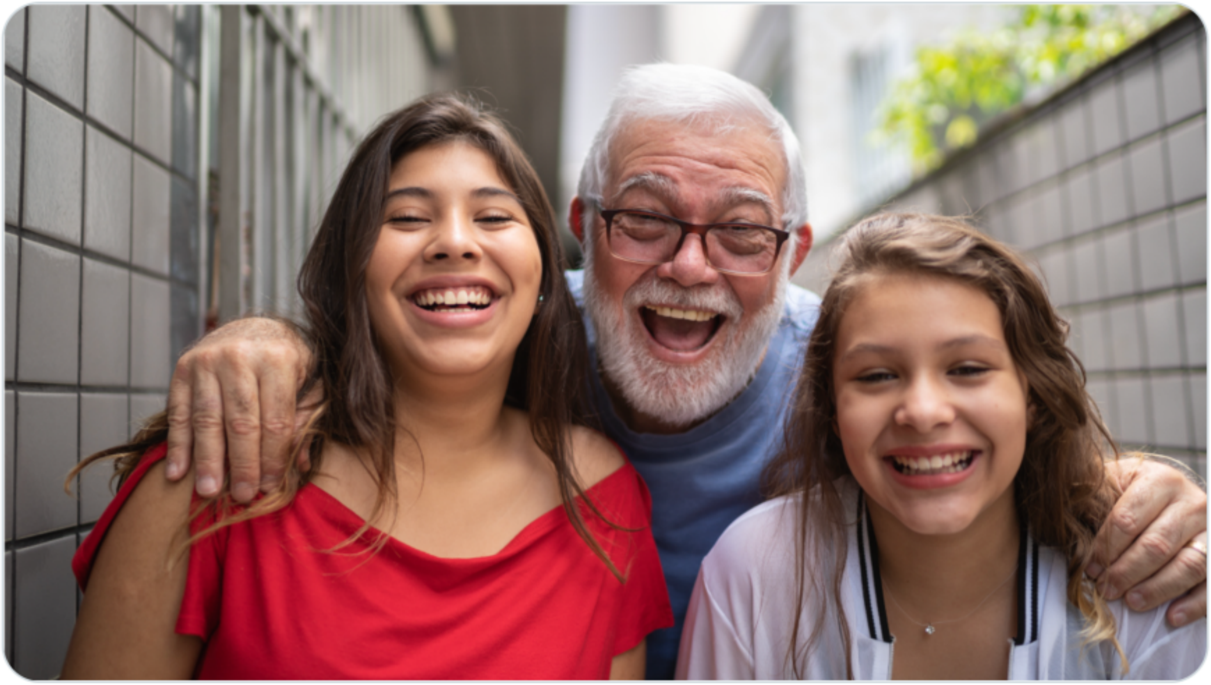

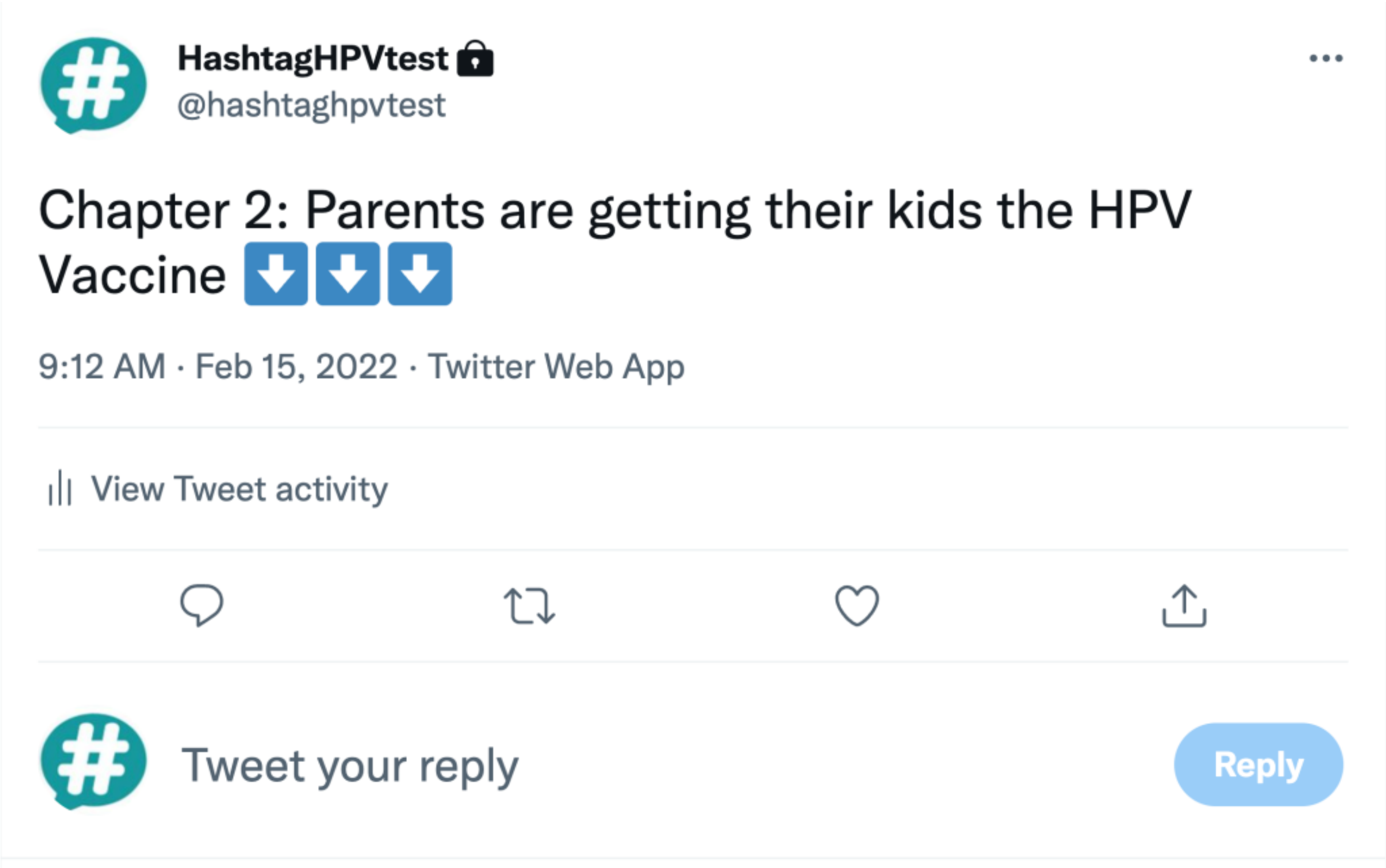
HashtagHPVtest
@hashtaghpvtest
Chapter 2: Parents are getting their kids the HPV Vaccine ⬇️⬇️⬇️
9:12 AM · Feb 15, 2022 · Twitter Web App
View Tweet activity
Tweet your reply
Reply

HashtagHPVtest 🔒 @hashtaghpvtest · Feb 15

Replying to @hashtaghpvtest

👩‍💻Jessica⚽: Our ped suggested bringing in our kids for the HPV vaccine & says most parents are doing it. My husband & I aren't sure about it...I've done serious HPV vaccine research & there's A LOT out there. Your kids also see Dr. Singh – what do you think? 1/7

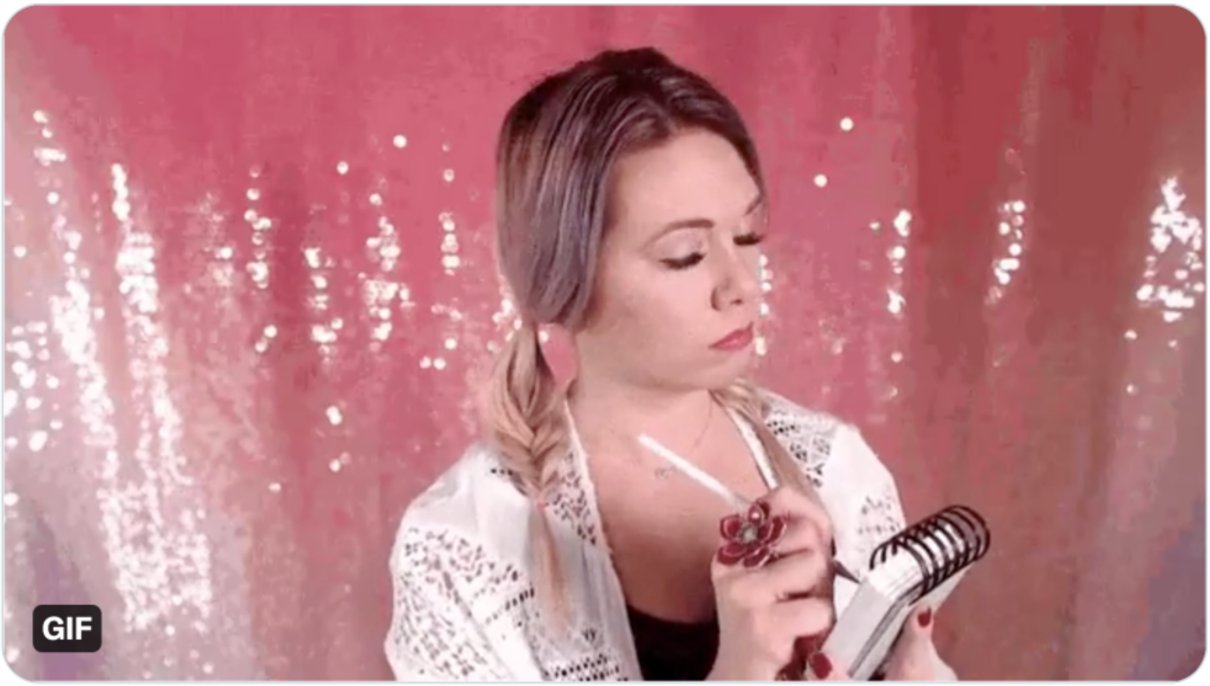

1

HashtagHPVtest @hashtaghpvtest · Feb 15

Dr. Singh
@drsingh

Most parents are choosing to get their children the HPV vaccine to protect them from cancers caused by HPV infections. Are your kids protected?

10:00 AM · May 24, 2021

1

HashtagHPVtest @hashtaghpvtest · Feb 15

👩🏽‍⚕️Lin🩺: Dr. Singh is right. A little over half of teens are getting the vax. Still not enough. 3/7

1

HashtagHPVtest 🔒 @hashtaghpvtest · Feb 15

👩🏾Dana☮️: A lot of anti-vax content out there. 😠 Let's listen to what the docs are saying. This is about protecting your kids from serious diseases like cancer. ALL parents SHOULD talk to their kids' doctor or nurse about the HPV vaccine. 4/7

1

HashtagHPVtest 🔒 @hashtaghpvtest · Feb 15

👩🏻‍⚕️Lin🩺: Misinformation is a big problem. Not enough info is probably a bigger problem. Trust the science! Here is some intel I found helpful and now share with my friends and patients: ow.ly/Otyf30fPkYT 5/7

1

HashtagHPVtest 🔒 @hashtaghpvtest · Feb 15

👩🏻‍💻Jessica⚽: Misinformation IS a big problem. I started my blog when my kids were young because of all the conflicting parenting advice I found! I hope to be a source for parents to find reliable information. I fact check all that I can. 6/7

1

HashtagHPVtest 🔒 @hashtaghpvtest · Feb 15

👴🏾Miguel🛻: I would trust Dr. Singh. She knows what she's talking about. My kids didn't get vaccinated, so they weren't protected. I'm happy I can keep my grandkids safe with the HPV vaccine – look how sweet they are! Proud abuelo 🥰 7/7

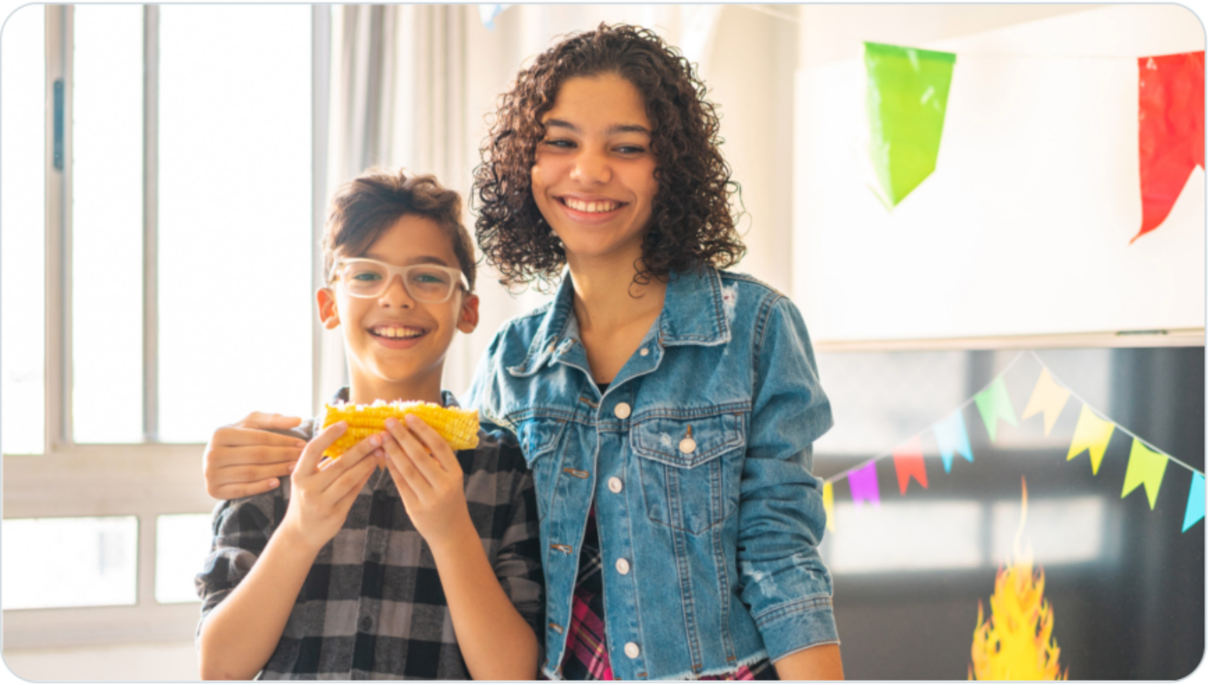

HashtagHPVtest 🔒
@hashtaghpvtest

Chapter 3: Learning more about the HPV Vaccine ⬇️⬇️⬇️

9:21 AM · Feb 16, 2022 · Twitter Web App

View Tweet activity

Tweet your reply

Reply

HashtagHPVtest 🔒 @hashtaghpvtest · Feb 16

Replying to @hashtaghpvtest

👴🏾Miguel🛻: When Dr. Singh recommended the HPV vaccine for my grandkids, I learned how common HPV is – most people don't even know they have it! ¡Qué pena! All these people are at risk for the diseases HPV can cause. Glad this info is more available today than it used to be. 1/9

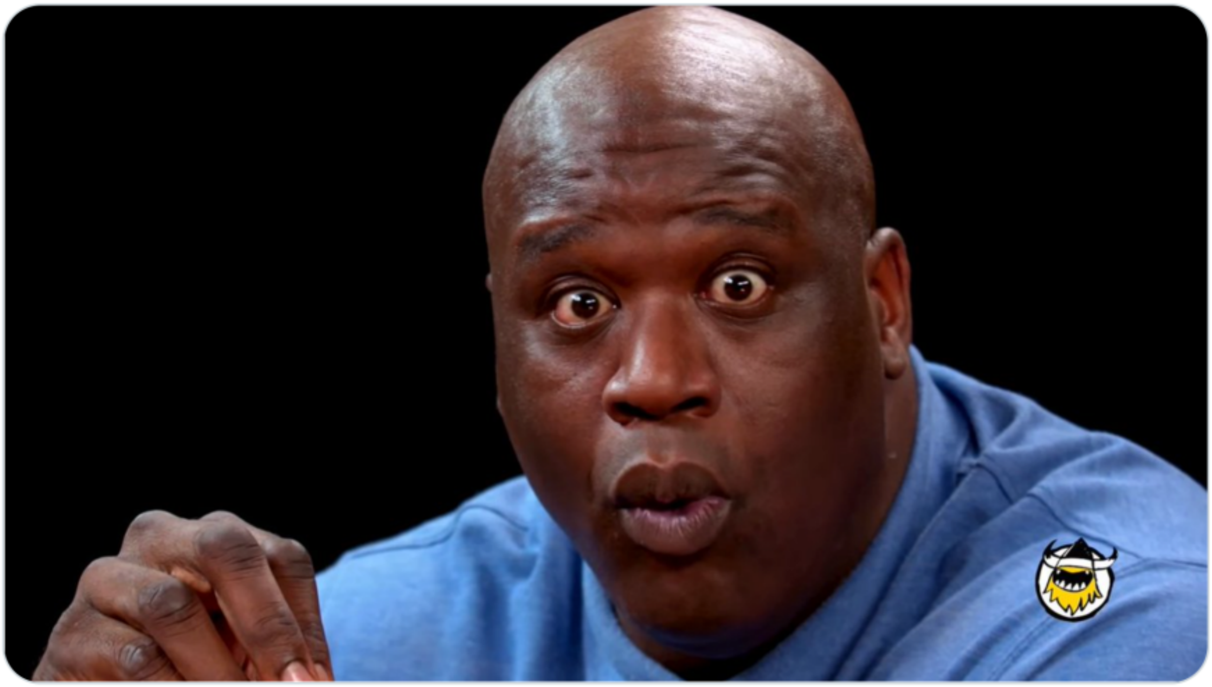

1

HashtagHPVtest 🔒 @hashtaghpvtest · Feb 16

👩🏻‍💻Jessica⚽: I've read that 85% of people in the U.S. will have HPV at some point in their lives. But then I also read that it usually goes away on its own. So, what's the story? Why do we even need the vaccine? 2/9

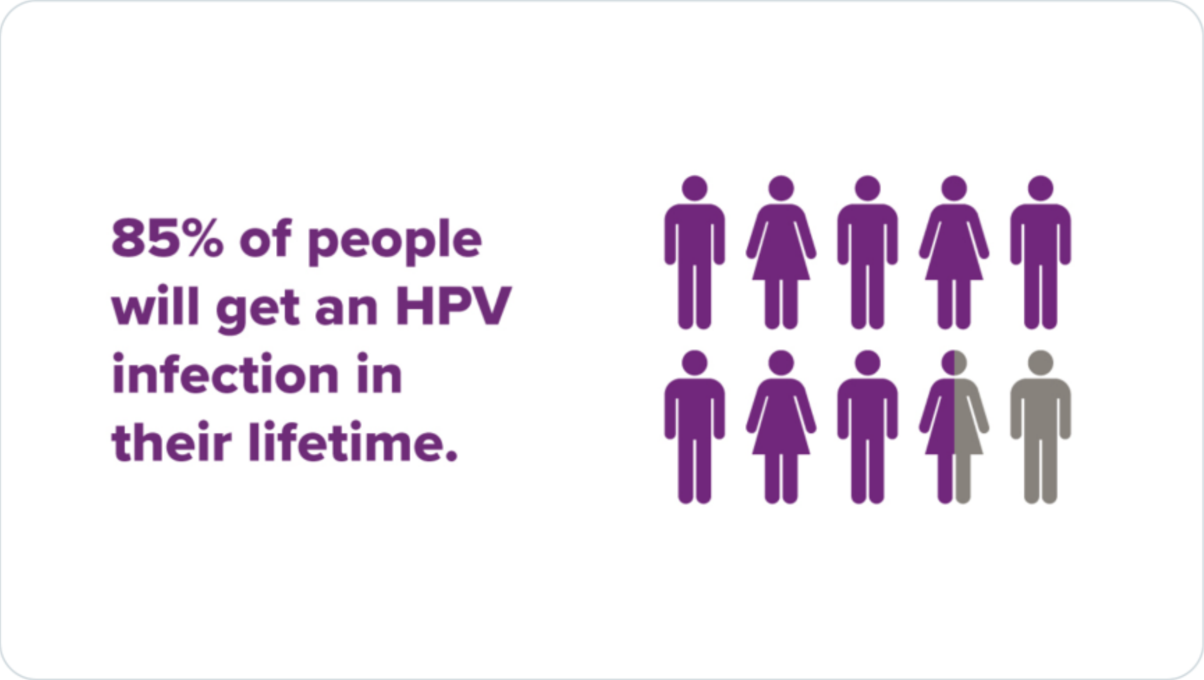

1

HashtagHPVtest 💼 @hashtaghpvtest · Feb 16

👩🏽‍⚕️Lin🩺: That's a question many parents have. I used to wonder the same thing. I've learned that while most people get HPV and
rarely have symptoms, there's no way to know which people who get HPV will develop cancer. 3/9

1

HashtagHPVtest 💼 @hashtaghpvtest · Feb 16

👩🏽‍⚕️Lin🩺: And the HPV vaccine protects against the most dangerous kinds of HPV – including the kinds that can cause cancer! That's why kids should get the HPV vaccine at 11 or 12, at the same time as their meningococcal and Tdap vaccines.4/9

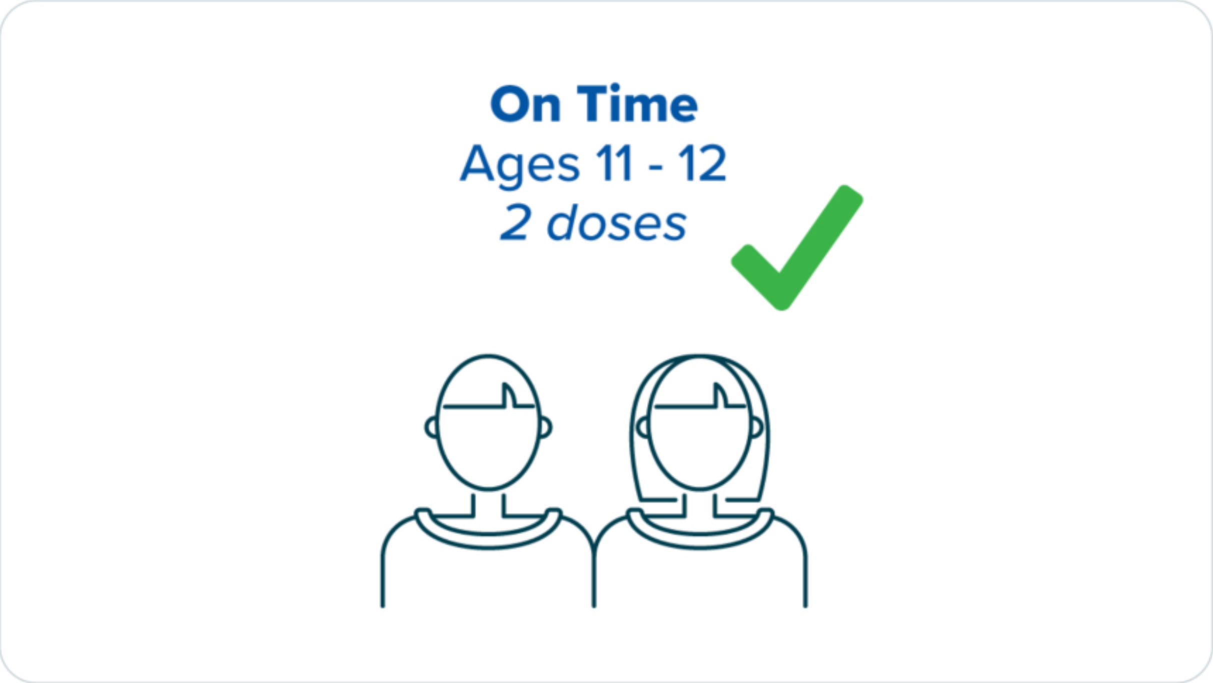

1

HashtagHPVtest 🔒 @hashtaghpvtest · Feb 16

👩‍💻Jessica⚽: It's safe to get all those shots at the same appointment? Doesn't that overwhelm their young immune systems? And I cringe thinking about how my oldest would do...she and her dad are both needle-phobes!5/9

1

HashtagHPVtest 🔒 @hashtaghpvtest · Feb 16

👩‍⚕️Lin🩺: Completely safe. A lot of research goes into determining when kids should get their vaccines. It's all about getting those shots to work safely and most effectively with kids' immune systems.6/9

1

HashtagHPVtest 🔒 @hashtaghpvtest · Feb 16

👩‍⚕️Lin🩺: And as a busy mom, being able to get those vaccines together is something I'm grateful for. Fewer doctor visits = 🙌 Think how many things you'll be protecting your daughter against in 1 appt! 7/9

1

HashtagHPVtest 🔒 @hashtaghpvtest · Feb 16

👩🏾Dana☮️: But don't forget to go back for her 2nd shot! I just took my daughter to get her 1st shot, and before we left the office, I made an appt for her 2nd. Here's my strong girl post-shot!8/9

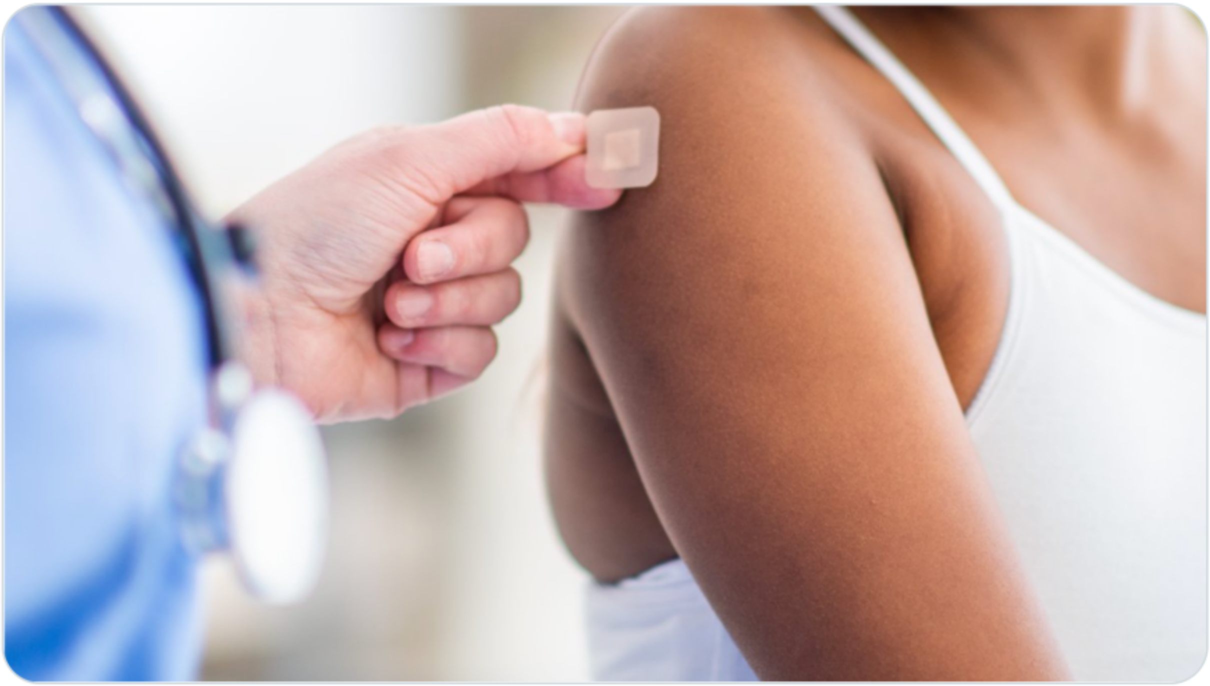

1

HashtagHPVtest @hashtaghpvtest · Feb 16

👩🏽‍⚕️ Lin🩺: Glad you're so on top of it. My son just got shot 2. And you bring up another reason kids should get the HPV vax at 11 or 12 – at that age, they only need 2 shots for complete protection. If they wait until they're older to start the vax series, they need 3! 9/9

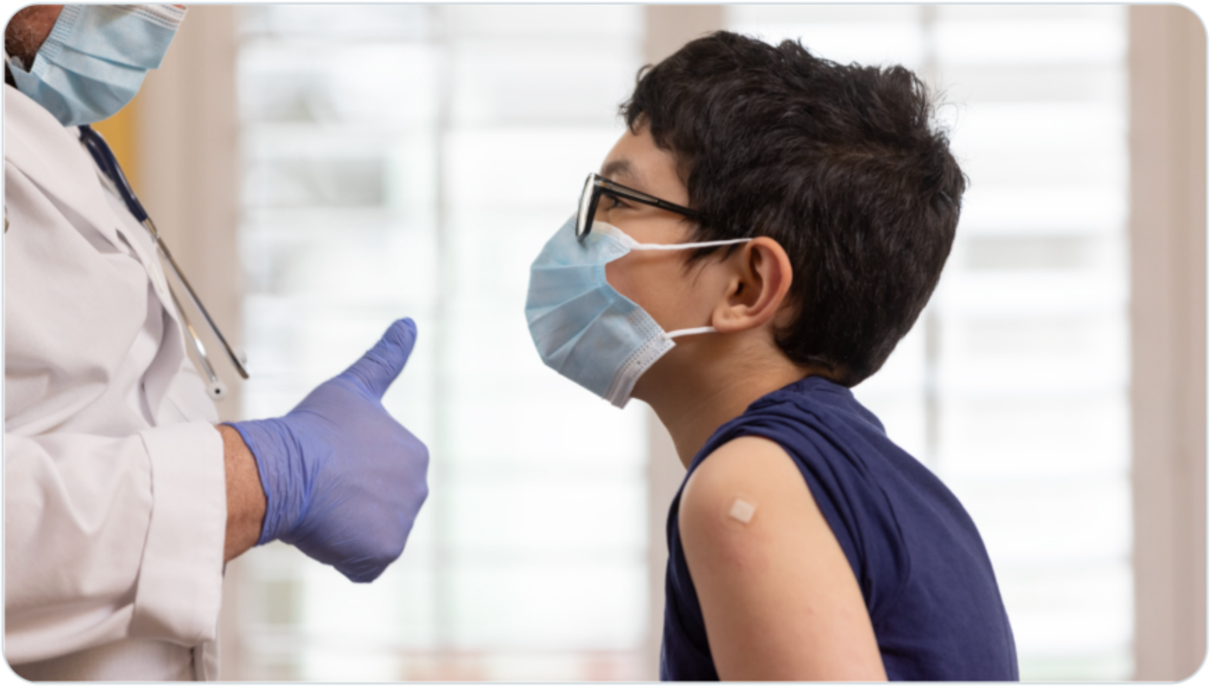

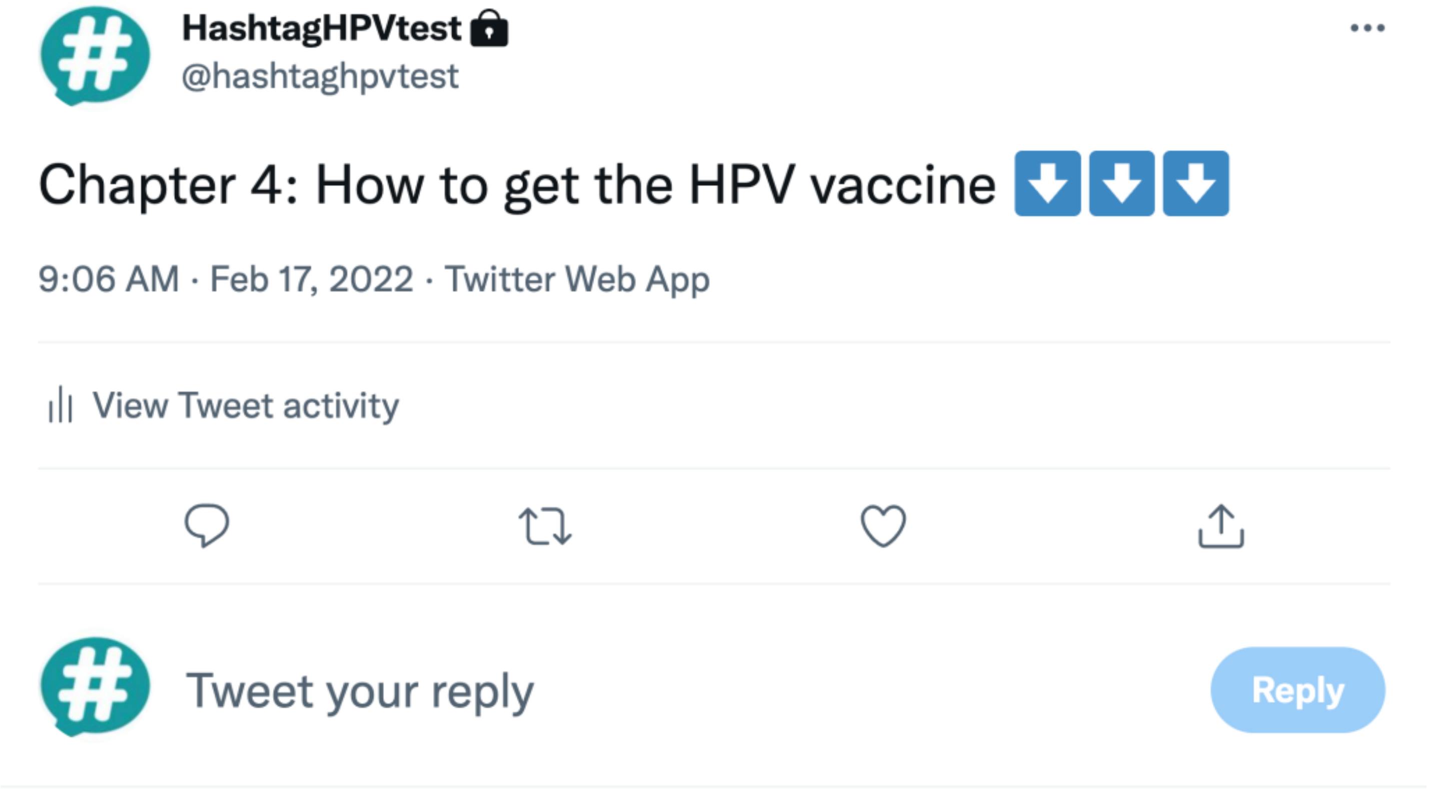
HashtagHPVtest
@hashtaghpvtest
Chapter 4: How to get the HPV vaccine ⬇️⬇️⬇️
9:06 AM · Feb 17, 2022 · Twitter Web App
View Tweet activity
Tweet your reply
Reply

HashtagHPVtest 🔒 @hashtaghpvtest · Feb 17
Replying to @hashtaghpvtest
👩‍💻Jessica⚽: Looking into the HPV vax a little more and trying to figure out when I could take my daughter in for her vaccine, if we do get it. I saw online that some docs offer evening and weekend hours – does Dr. Singh do this?1/9

1

HashtagHPVtest 🔒 @hashtaghpvtest · Feb 17
👩🏾Dana☮️: Yep, I snagged a Saturday appt when scheduling my daughter's second shot. 2/9

1

HashtagHPVtest 🔒 @hashtaghpvtest · Feb 17
👴🏽Miguel🛻: I know Dr. Singh has some evening appts, too. I took one of my grandkids to get his. He felt fine after and it was no big deal. Don't forget grandparents can help! I was glad to do so, and my son was glad to have one less thing on his plate. 3/9

1

HashtagHPVtest 🔒 @hashtaghpvtest · Feb 17
👩‍💻Jessica⚽: Good to know. The flexibility is nice for other ped appointments, too. I should put that on my blog – my readers are always looking for hacks to fit things into their busy days. 4/9

1

HashtagHPVtest 🔒 @hashtaghpvtest · Feb 17

👩‍💻Jessica⚽: I also read that the HPV vaccine is available for free. Is that true? Did insurance cover the vaccine for you? 5/9

1

HashtagHPVtest 🔒 @hashtaghpvtest · Feb 17

👩‍⚕️ Lin🩺: Yes! The HPV vaccine is free with most insurance plans.6/9

1

HashtagHPVtest 🔒 @hashtaghpvtest · Feb 17

👩🏾Dana☮️: Even without insurance too! There's a program for uninsured and Medicaid-eligible kids through age 18 to get free vaccines called Vaccines for Children. Check it out: bit.ly/3eysGxu 7/9

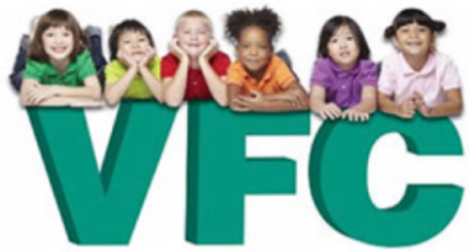

1

HashtagHPVtest 🔒 @hashtaghpvtest · Feb 17

👩‍⚕️ Lin🩺: The hospital is setting up a mobile clinic to give out free vaccines through VFC. I volunteered to get that started – I've got lots of experience working at free clinics from back in the day.8/9

1

HashtagHPVtest 🔒 @hashtaghpvtest · Feb 17

👩‍💻Jessica⚽: Very cool, Lin! And thanks, everyone. Good to know insurance plans cover vaccination. And so great that there are options for kids to get the HPV vaccine for free, even without insurance!9/9

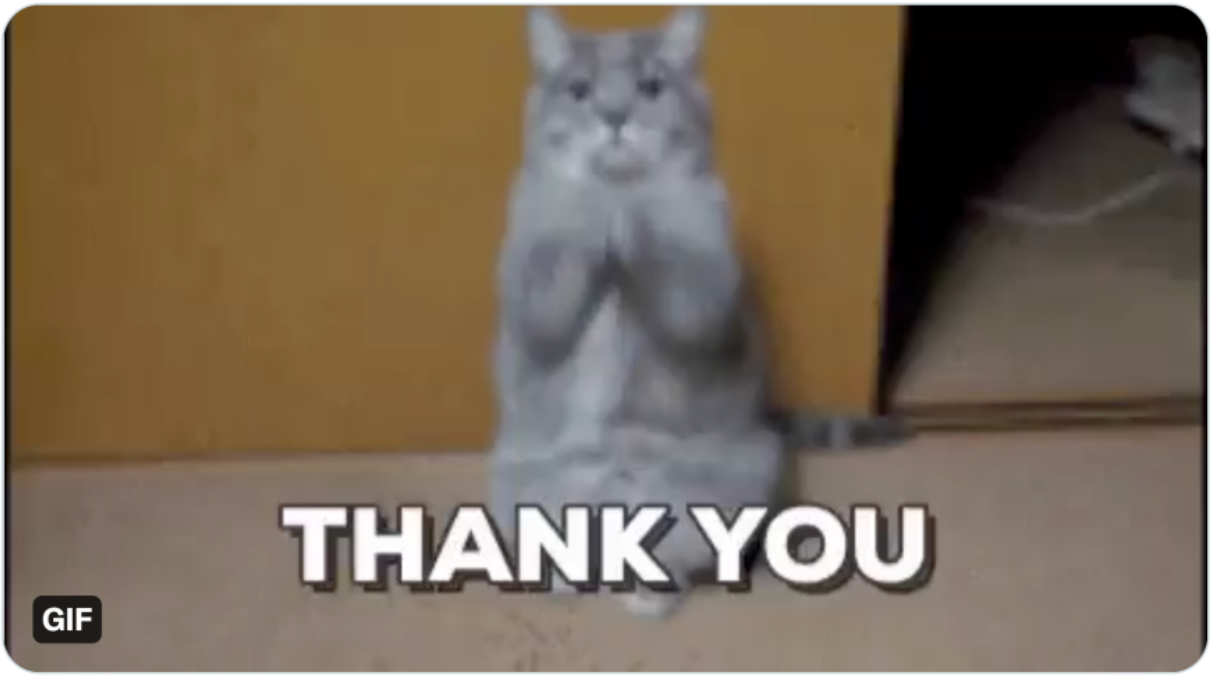

HashtagHPVtest 🔒
@hashtaghpvtest

Chapter 5: Did you know the HPV vaccine has been around for longer than most of us have been parents ⬇️⬇️⬇️

9:09 AM · Feb 21, 2022 · Twitter Web App

View Tweet activity

Tweet your reply

Reply

HashtagHPVtest @hashtaghpvtest · Feb 21

Replying to @hashtaghpvtest

Dana: One of my Facebook friends insisted she's keeping her daughter safe by NOT getting the HPV vaccine. There's so many facts that prove the opposite. Vaccines will KEEP her daughter safe. I shared this page with her...here's hoping she reads it! bit.ly/3fWiUou 1/8

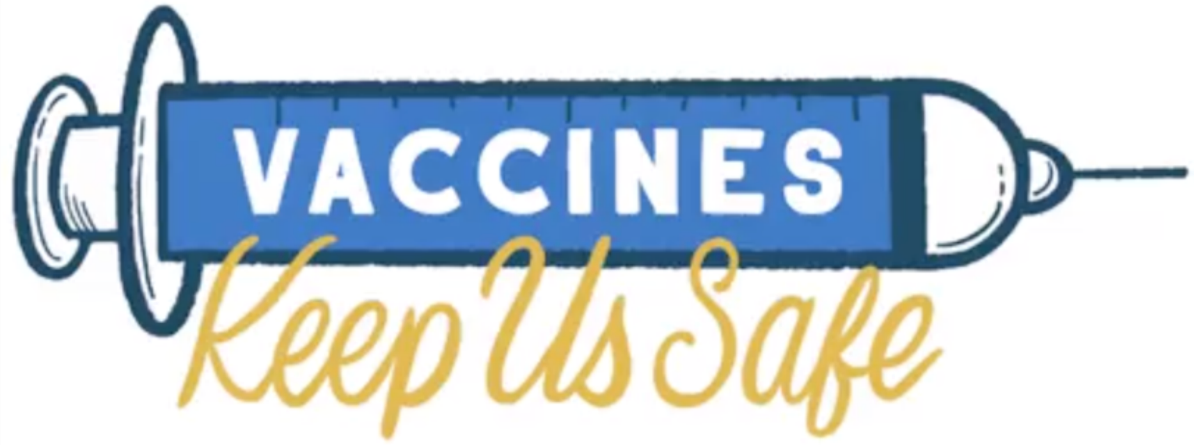

GIF

1

HashtagHPVtest 🔒 @hashtaghpvtest · Feb 21

👩🏻‍💻Jessica⚽: I've seen so many questions about vaccine safety on blogs and forums, and there's a lot of misinformation out there. I feel for all us parents – tons of choices to make when raising kids. We just want to do the right thing to keep our families healthy and safe. 2/8

1

HashtagHPVtest 🔒 @hashtaghpvtest · Feb 21

👩🏾Dana☮️: I get the concern about making the right choice, but Dr. Singh wouldn't recommend it if it wasn't safe. My daughter was surprised when I told her I got the vaccine & that it was 3 shots back in the day. 😯 She's not scared of shots, but still, 2 is easier than 3! 3/8

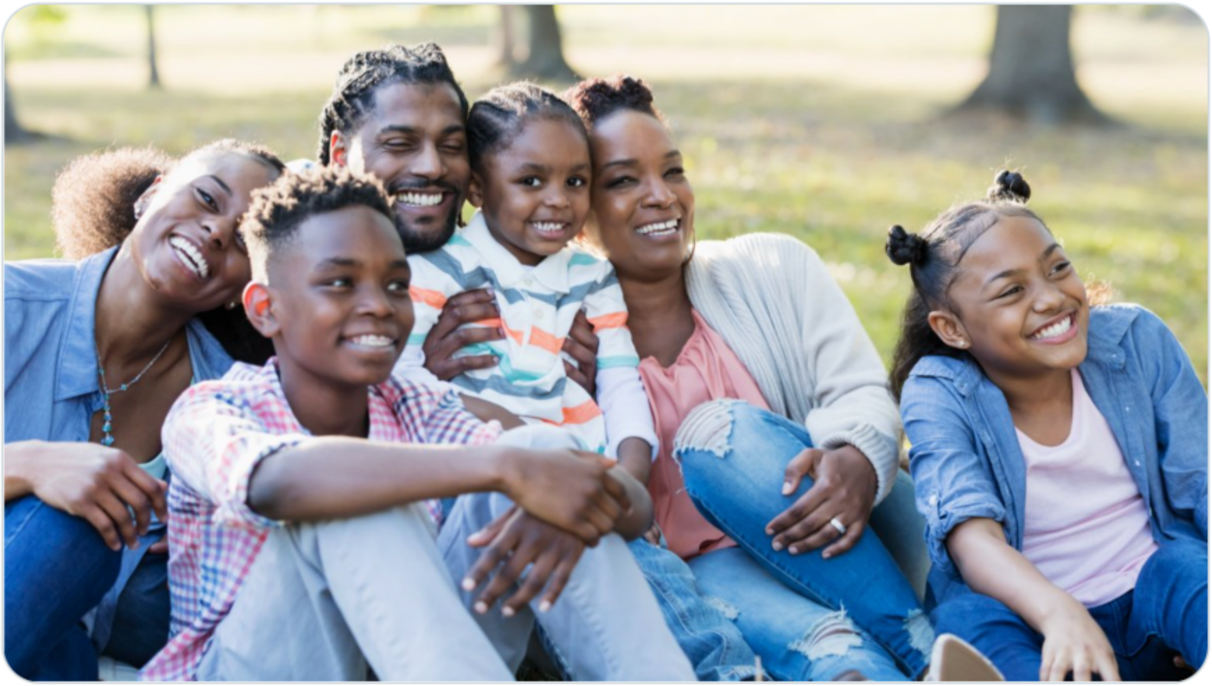

1

HashtagHPVtest 🔒 @hashtaghpvtest · Feb 21

👩🏽‍⚕️Lin🩺: Dana's right: All vaccines go through rigorous testing to make sure they're safe and effective. Same with the HPV vaccine. It's been monitored for safety for 15 years. I remember it being a topic back when I was working on my master's degree in public health.4/8

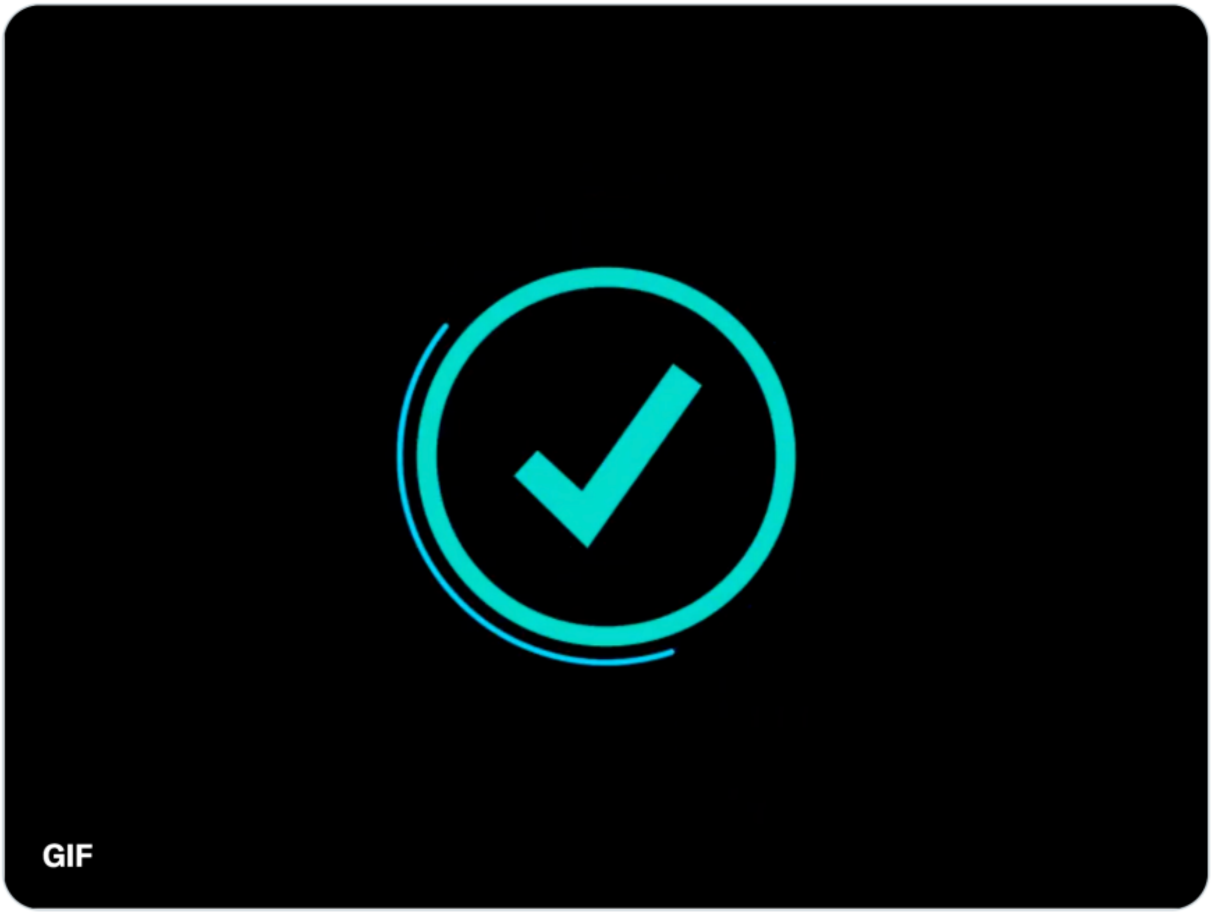

1

HashtagHPVtest 🔒 @hashtaghpvtest · Feb 21

👩🏻‍💻Jessica⚽: 15 years – that's longer than I've been a mom! My husband is a still little skeptical, but I'm with you both – I've asked Dr. Singh all about the safety of the vaccine and have been reassured that it's safe and effective.5/8

1

HashtagHPVtest 🔒 @hashtaghpvtest · Feb 21

👴🏾Miguel🛻: Safe and pretty painless, too – nothing a fun bandage and an ice cream cone couldn't fix! Here's Nicolas enjoying his treat after his shot. Okay, so abuelo wanted an excuse to get ice cream…but my grandson didn't complain. 😊6/8

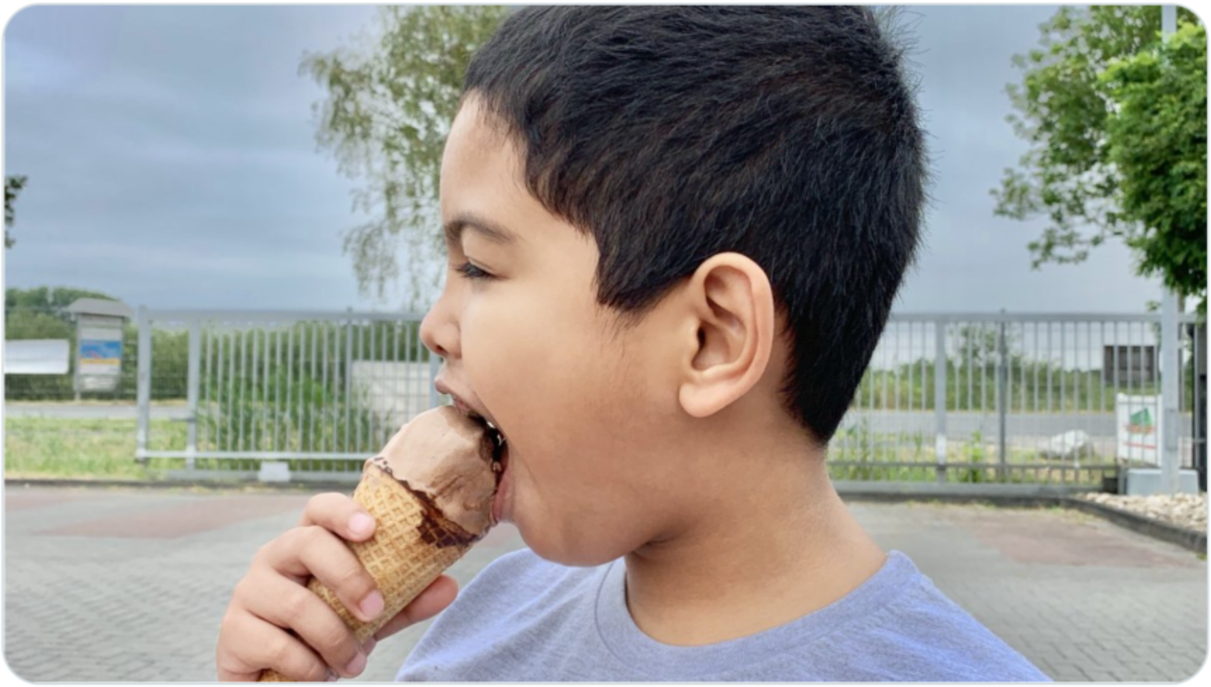

1

HashtagHPVtest 🔒 @hashtaghpvtest · Feb 21

👩‍💻Jessica⚽: What about now that kids who are 12 or older can get the COVID-19 vaccine? Is it safe to get the HPV vaccine and COVID-19 vaccine at the same time? Will the vaccines still work?7/8

1

HashtagHPVtest 🔒 @hashtaghpvtest · Feb 21

👩‍⚕️Lin🩺: This has come up a lot lately at the hospital where I work. We got a memo saying it's safe to give a COVID-19 vaccine at the same time as others, like the HPV vaccine. They still work just as well. Dr. Singh will say the same. Here's some info:bit.ly/3yXOoU6 8/8

HashtagHPVtest 🔒
@hashtaghpvtest

Chapter 6: Boys and girls can both get the HPV vaccine ⬇️⬇️⬇️

9:11 AM · Feb 22, 2022 · Twitter Web App

View Tweet activity

Tweet your reply

Reply

HashtagHPVtest 🔒 @hashtaghpvtest · Feb 22

Replying to @hashtaghpvtest

👩🏽‍⚕️ Lin🩺: 1 thing I hear a lot at the hospital I work at is many parents ask about the HPV vaccine for their daughters but not for their sons. Miguel, I'm glad your grandson got the vaccine. Boys can get HPV cancers, too, so they need the vaccine just as much as girls do. 1/6

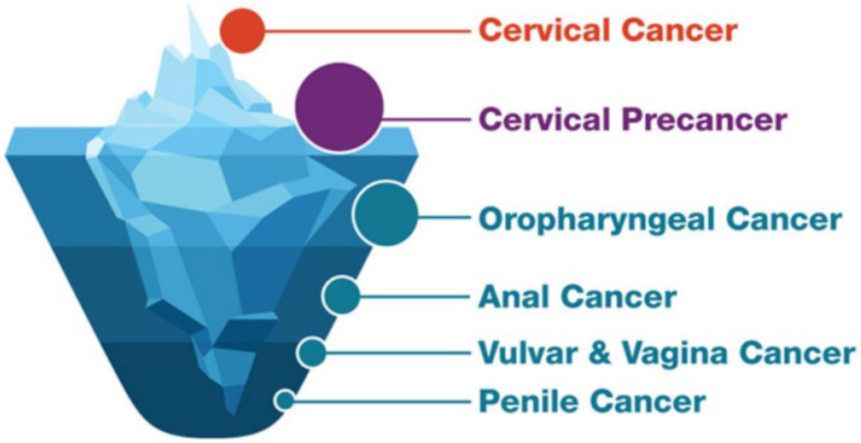

1

HashtagHPVtest 🔒 @hashtaghpvtest · Feb 22 ···

👩‍💻Jessica⚽: Lin, you inspired me. In my search on HPV and boys, I learned almost ½ of cancers from HPV occur in men! Found that here bit.ly/3ccvGye. More good facts before I vax. When my daughter gets her shot, I'll ask about getting my son vaccinated, too.2/6

1

HashtagHPVtest 🔒 @hashtaghpvtest · Feb 22 ···

👴🏾Miguel🛻: The vaccine has changed over the years. And so have attitudes. Now the HPV vaccine is as much about protecting boys as girls. It protects against more cancers today too. Bring it up with Dr. Singh, she knows the history of the HPV vaccine.3/6

1

HashtagHPVtest 🔒 @hashtaghpvtest · Feb 22 ···

👴🏾Miguel🛻: This is hard for me to share...but my son got throat cancer a few years ago. His dentist found a lump...they caught it and treated it early enough. Gracias a Dios. But we were so scared. His doctor said throat cancer is one of the most common types of HPV cancer. 4/6

1

HashtagHPVtest 🔒 @hashtaghpvtest · Feb 22 ···

👩🏾Dana☮️: I can't imagine my daughter having a scare like that – so sorry to hear you and your family had to go through that! 5/6

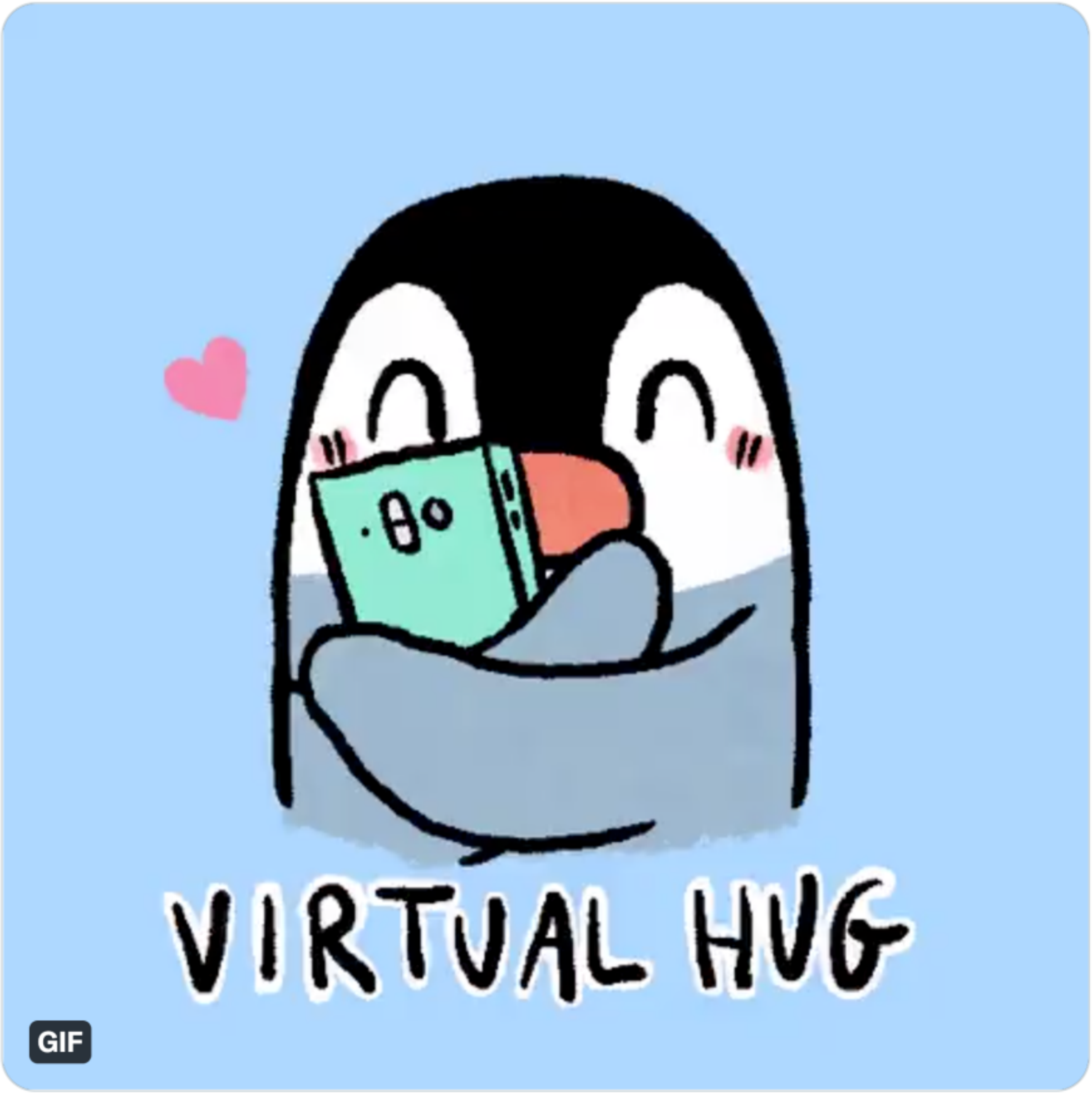

1

HashtagHPVtest 🔒 @hashtaghpvtest · Feb 22 ···

👩🏻‍💻Jessica⚽: I've read about way too many similar stories shared online. I'm glad all turned out okay, and thankful there is a vaccine now to protect our kids and Miguel's grandkids. 6/6

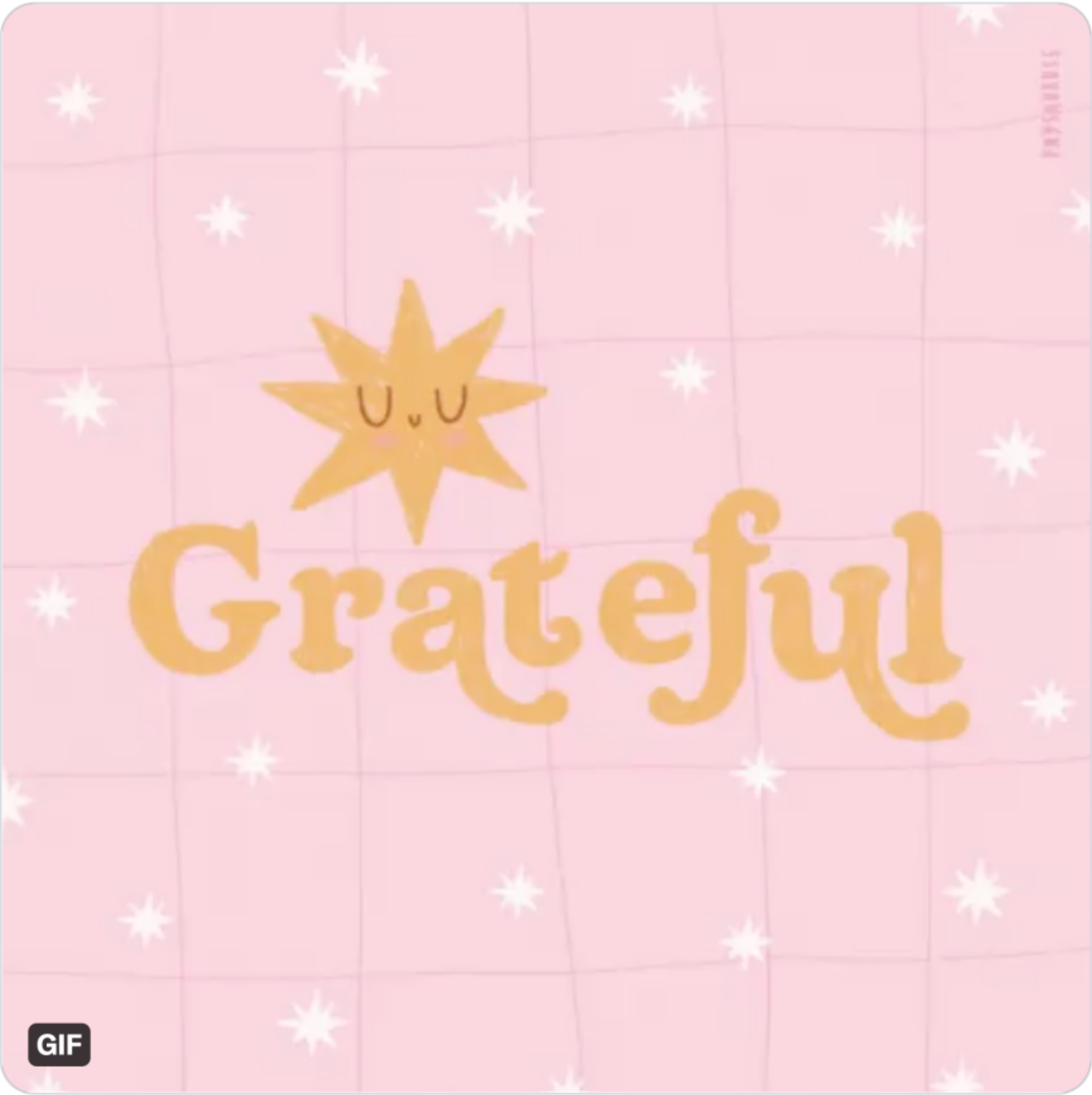
Grateful
GIF

HashtagHPVtest 🔒
@hashtaghpvtest

Chapter 7: All communities can benefit from the HPV vaccine ⬇️⬇️⬇️

9:01 AM · Feb 23, 2022 · Twitter for iPhone

View Tweet activity

Tweet your reply

Reply

HashtagHPVtest 🔒 @hashtaghpvtest · Feb 23
Replying to @hashtaghpvtest
👩🏾Dana☮️: Appreciation tweet for Dr. Singh, who brought up the HPV vaccine convo & is open to all my Qs. We switched from a ped where I had to push to get the same treatment as other kids for my daughter. I never feel like my race is a barrier to good care with Dr. Singh. 1/7

1

HashtagHPVtest 🔒 @hashtaghpvtest · Feb 23
👴🏽Miguel🛻: I've felt language can be a barrier at the doctor too. Spanish is my 1st language & I don't always follow what Dr. Singh's saying. But I'm not afraid to ask questions, & Dr. Singh is open about not just the check-up but my grandkids' overall health. 2/7

1

HashtagHPVtest 🔒 @hashtaghpvtest · Feb 23 ···

👴🏽Miguel🛻: In fact, when I took my grandson to get his vaccine, Dr. Singh told me Hispanic women have higher rates of cervical cancer, which is usually caused by HPV. ¡Ay! I think of my granddaughters…I'll make sure they're vaccinated and protected. 3/7

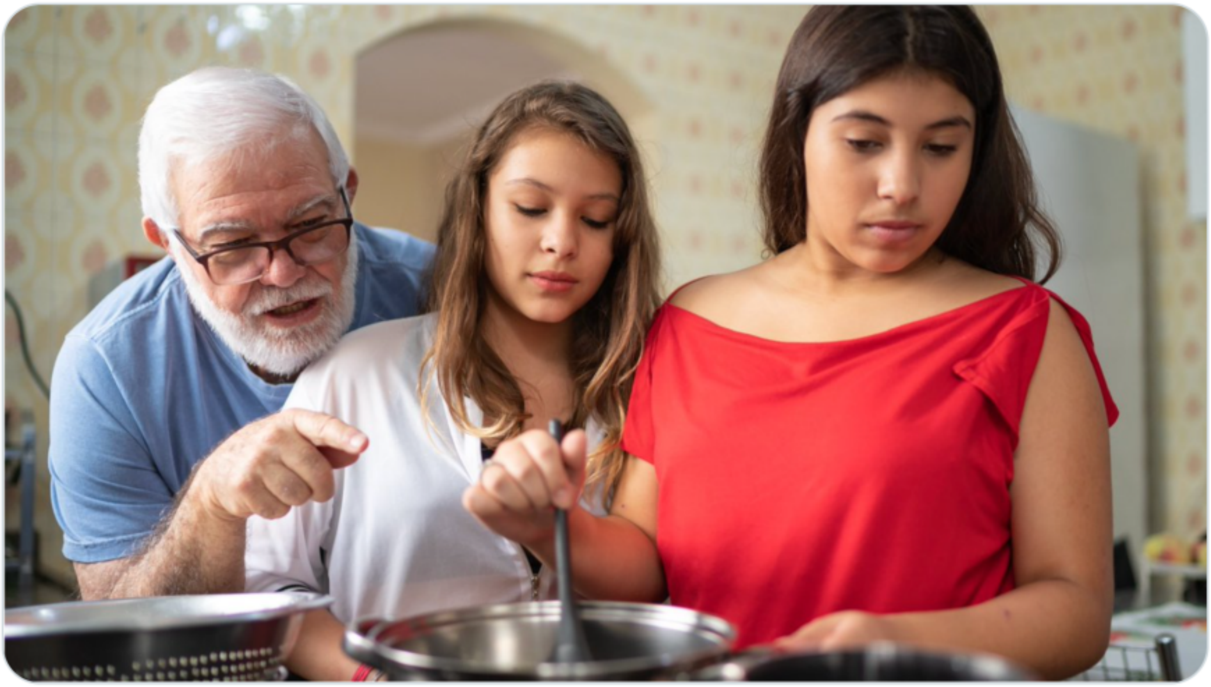

1

HashtagHPVtest 🔒 @hashtaghpvtest · Feb 23 ···

👩🏾Dana☮️: I told my friend who's a nurse that my daughter got the HPV vaccine, and she said Black women have the highest mortality rate from HPV-related cancers like cervical cancer. Makes me even
more determined to get my daughter dose 2 on time and fully protected ASAP.4/7

1

HashtagHPVtest 🔒 @hashtaghpvtest · Feb 23 ···

👩🏻‍⚕️Lin🩺: Dana & Miguel, the information you received is solid. I've seen while working in hospitals how some communities experience worse outcomes from HPV. Giving kids the HPV vaccine is the best way to change the story.5/7

1

HashtagHPVtest 🔒 @hashtaghpvtest · Feb 23 ···

👩🏾Dana☮️: It's a burden on Black women and one I feel personally – my grandma had cervical cancer and didn't have access to good health care. It was most likely from HPV, since that's the cause of most cervical cancers. 6/7

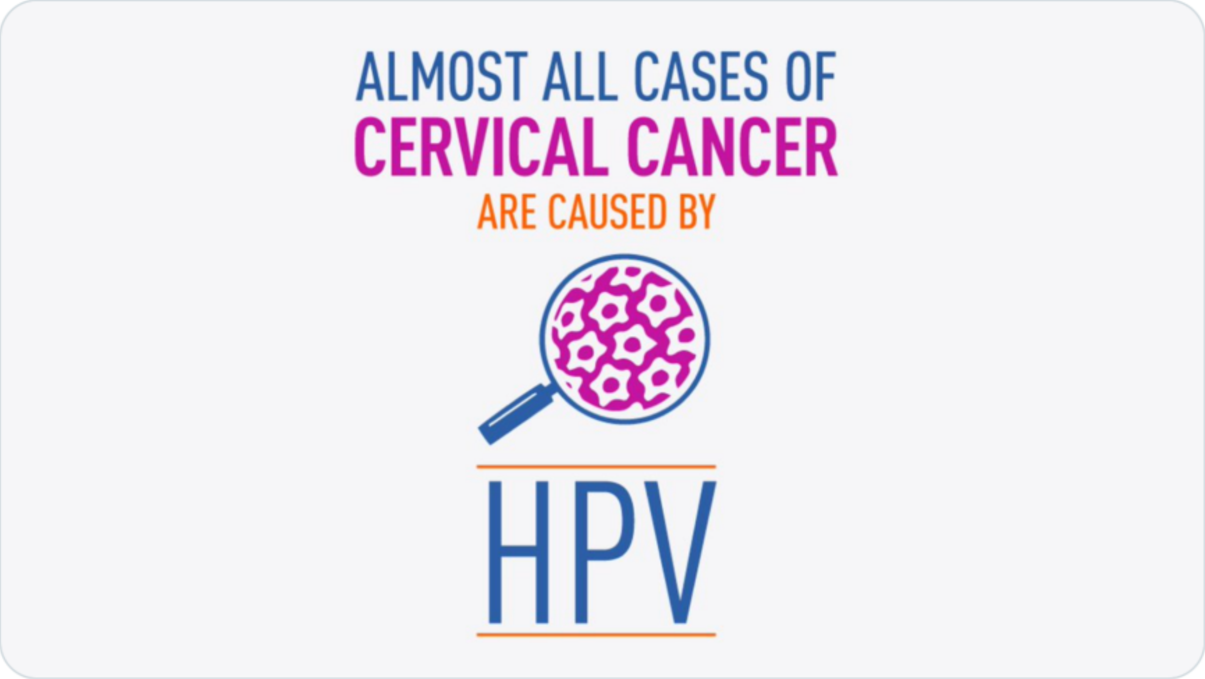

1

HashtagHPVtest 🔒 @hashtaghpvtest · Feb 23 ···

👩🏻‍💻Jessica⚽: So great that you're standing up for your families to get them the care they need. But I'm sorry that's needed. I think some people aren't aware of these barriers in different communities. I shared info on my blog to get my readers aware, too. ❤️7/7

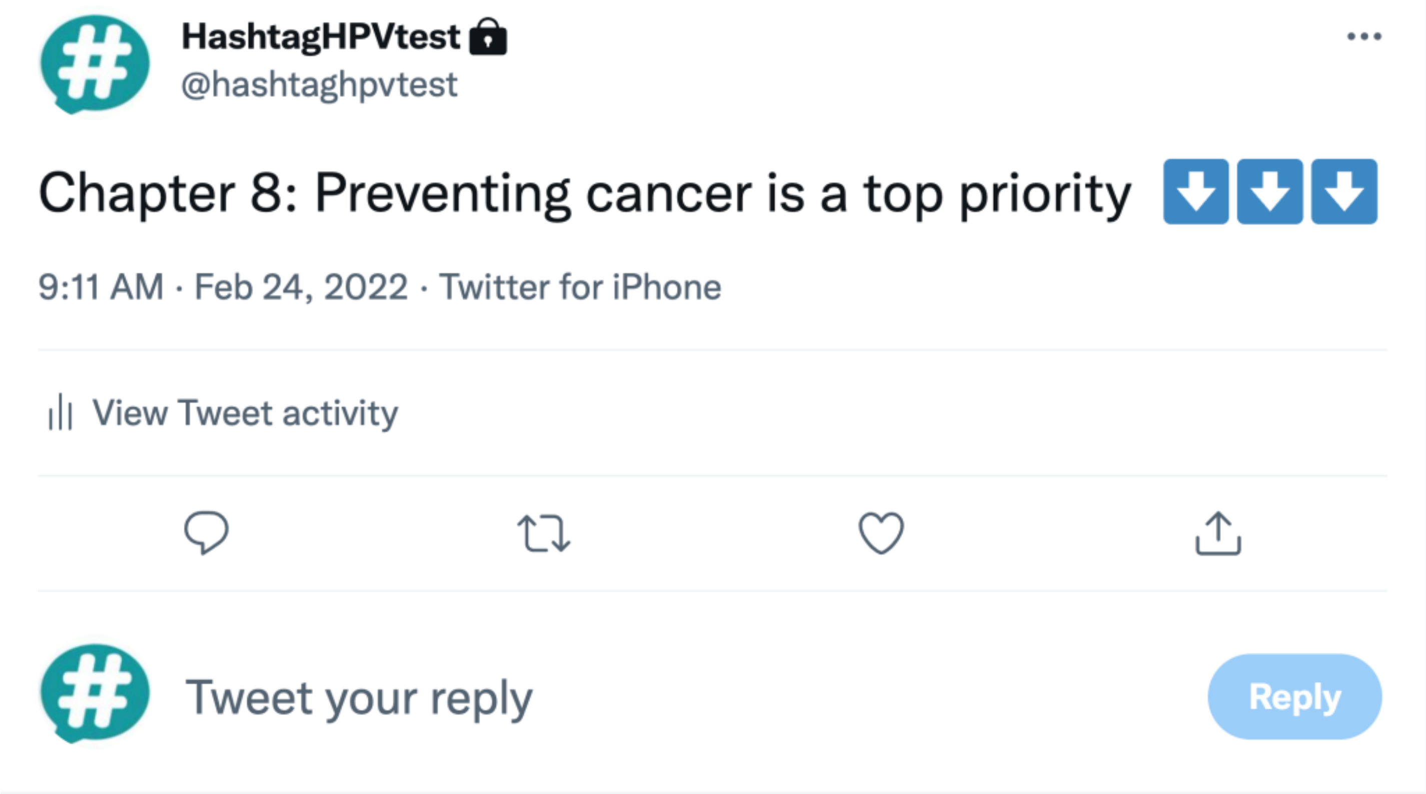
HashtagHPVtest
@hashtaghpvtest
Chapter 8: Preventing cancer is a top priority ⬇️⬇️⬇️
9:11 AM · Feb 24, 2022 · Twitter for iPhone
View Tweet activity
Tweet your reply
Reply

HashtagHPVtest 🔒 @hashtaghpvtest · Feb 24

Replying to @hashtaghpvtest

👩‍💻Jessica⚽: Ok don't judge, but you all know I've been on the fence about the HPV vaccine... Thinking about our convos and all my research has helped me decide to bring in my son & daughter for their first HPV vaccine shots! Here's my guy after.1/9

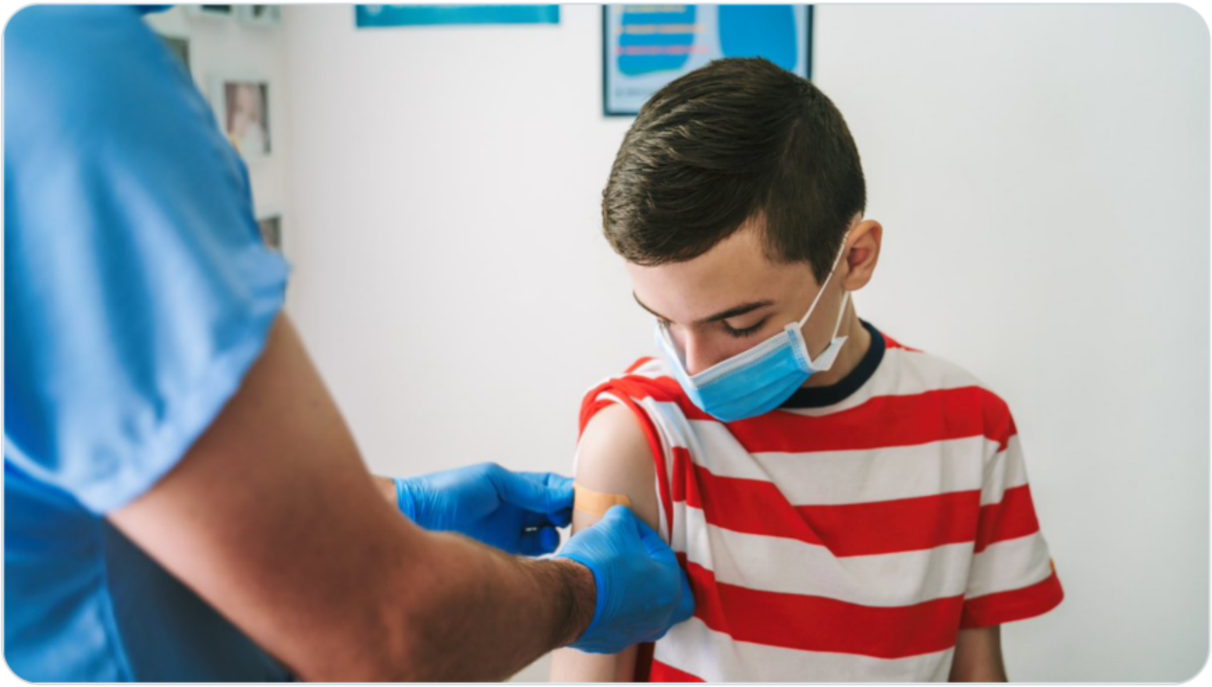

1

HashtagHPVtest 🔒 @hashtaghpvtest · Feb 24 ···

👩🏾Dana☮️: Way to go, Jessica! At the end of the day, you're protecting your kids against cancer.2/9

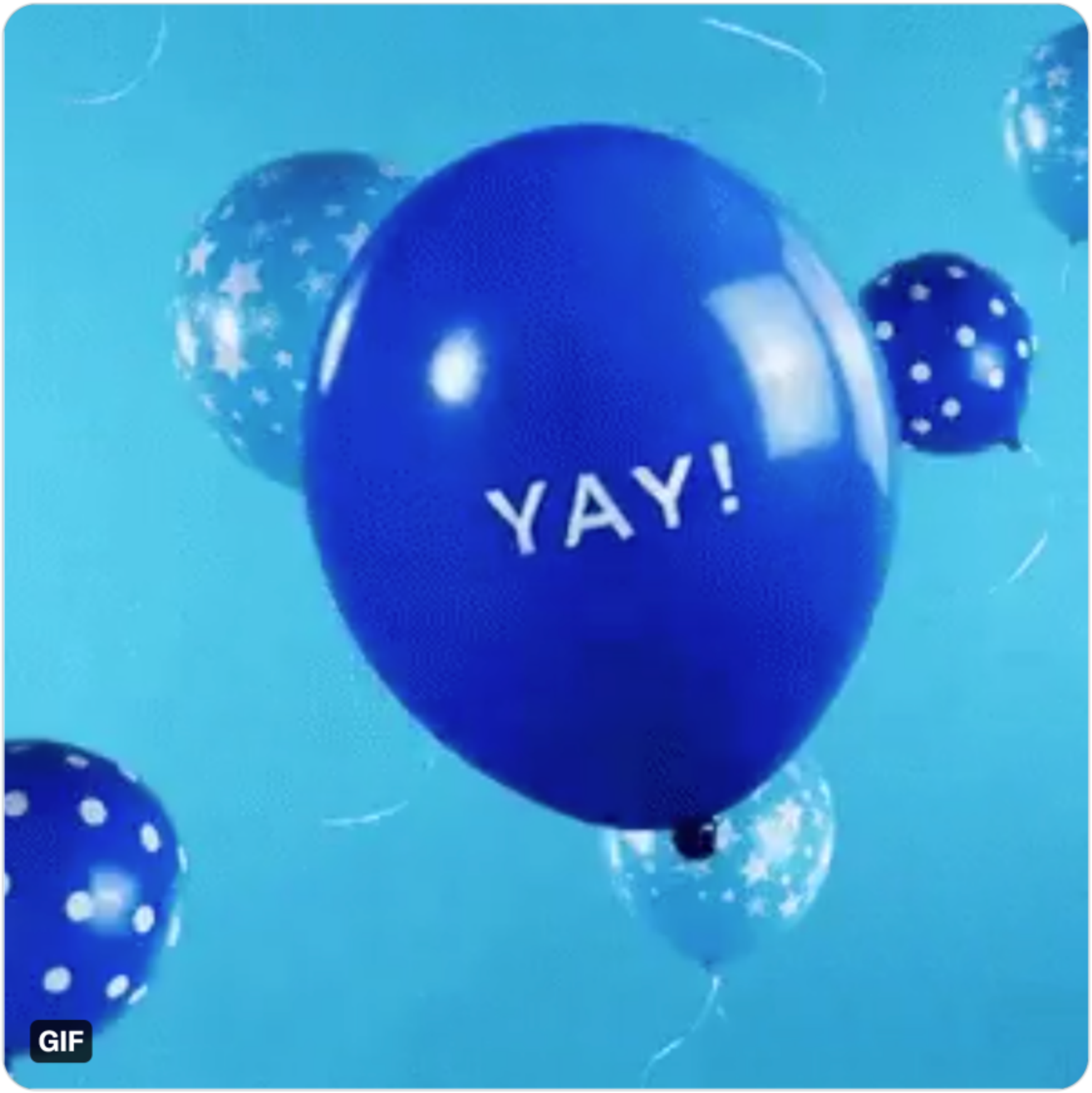

1

HashtagHPVtest @hashtaghpvtest · Feb 24

Lin: I almost waited to get my son his 1st HPV vaccine dose since he was already getting a couple other shots during his appt with Dr. Singh ... but I knew I couldn't put off starting the HPV vaccine – cancer won't wait until you're ready to take action.3/9

1

HashtagHPVtest @hashtaghpvtest · Feb 24

Jessica: Lin, I feel better hearing that even you almost took a pause. I was anxious about making the right decision...but it felt even worse to think about my kids getting a cancer that I could have helped prevent. 4/9

1

HashtagHPVtest @hashtaghpvtest · Feb 24

Lin: Yes! We all want to do what's best for our families. When I reminded myself that the HPV vaccine protects against 6 types of cancer, it was a no-brainer.5/9

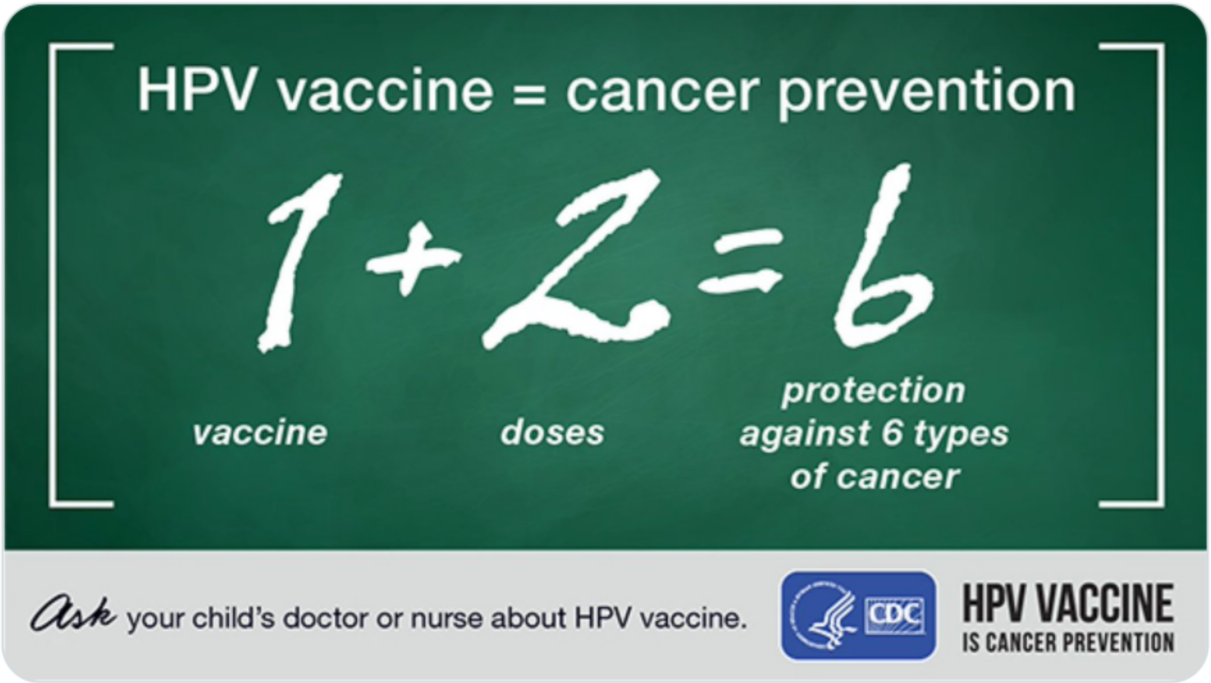

1

HashtagHPVtest @hashtaghpvtest · Feb 24

Miguel: When my son had his throat cancer, my own family was seriously impacted by HPV and I remember wishing I could have done more to prevent that. I'm relieved my grandkids will have a different story. 6/9

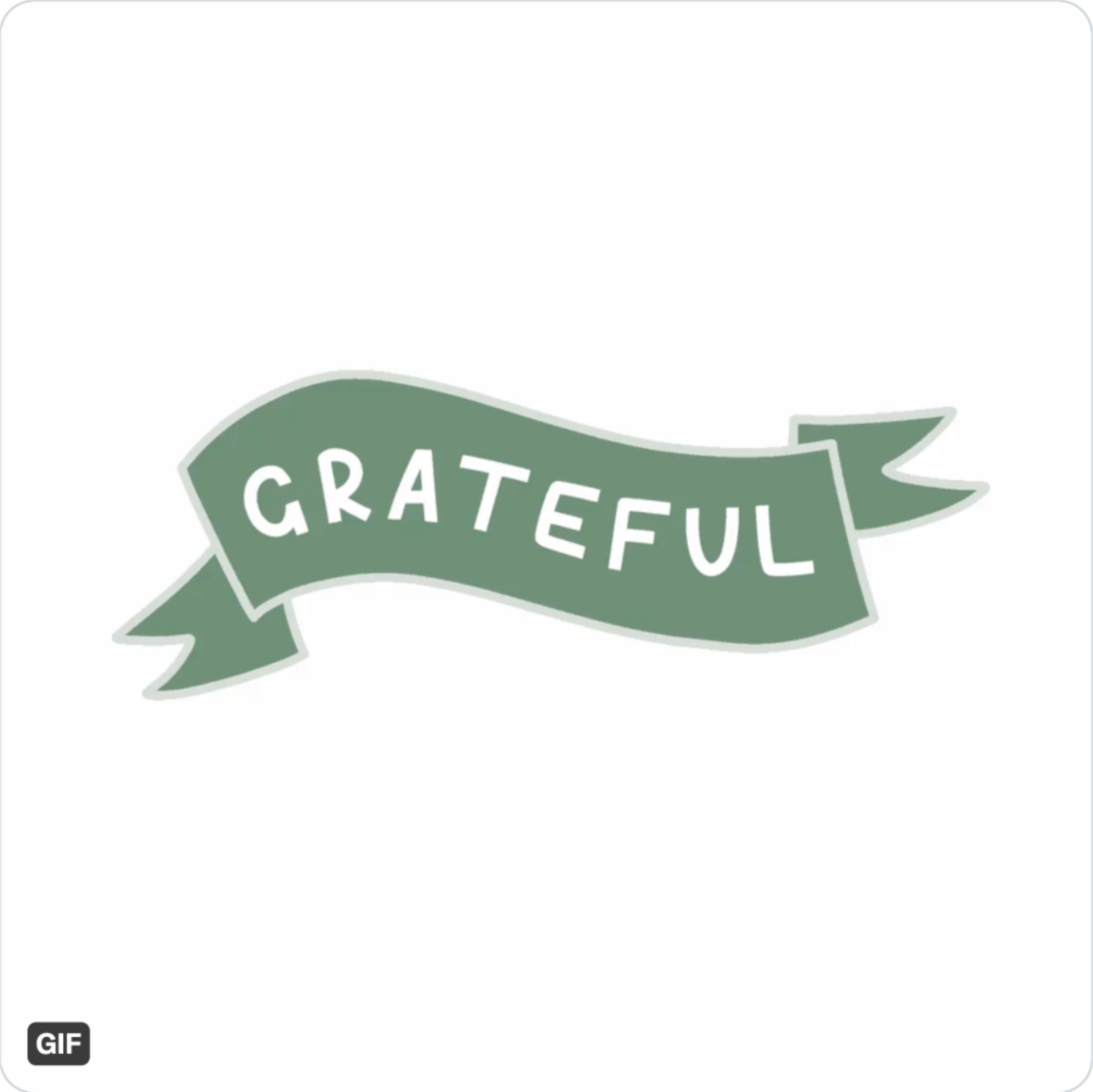

1

HashtagHPVtest @hashtaghpvtest · Feb 24

Jessica: I love this judgment-free zone! It's refreshing to share concerns & ask questions with no shame. We're all parents & want the best for our kids. Raising tweens can be confusing & stressful...I'm glad choosing whether to get the HPV vaccine doesn't have to be.7/9

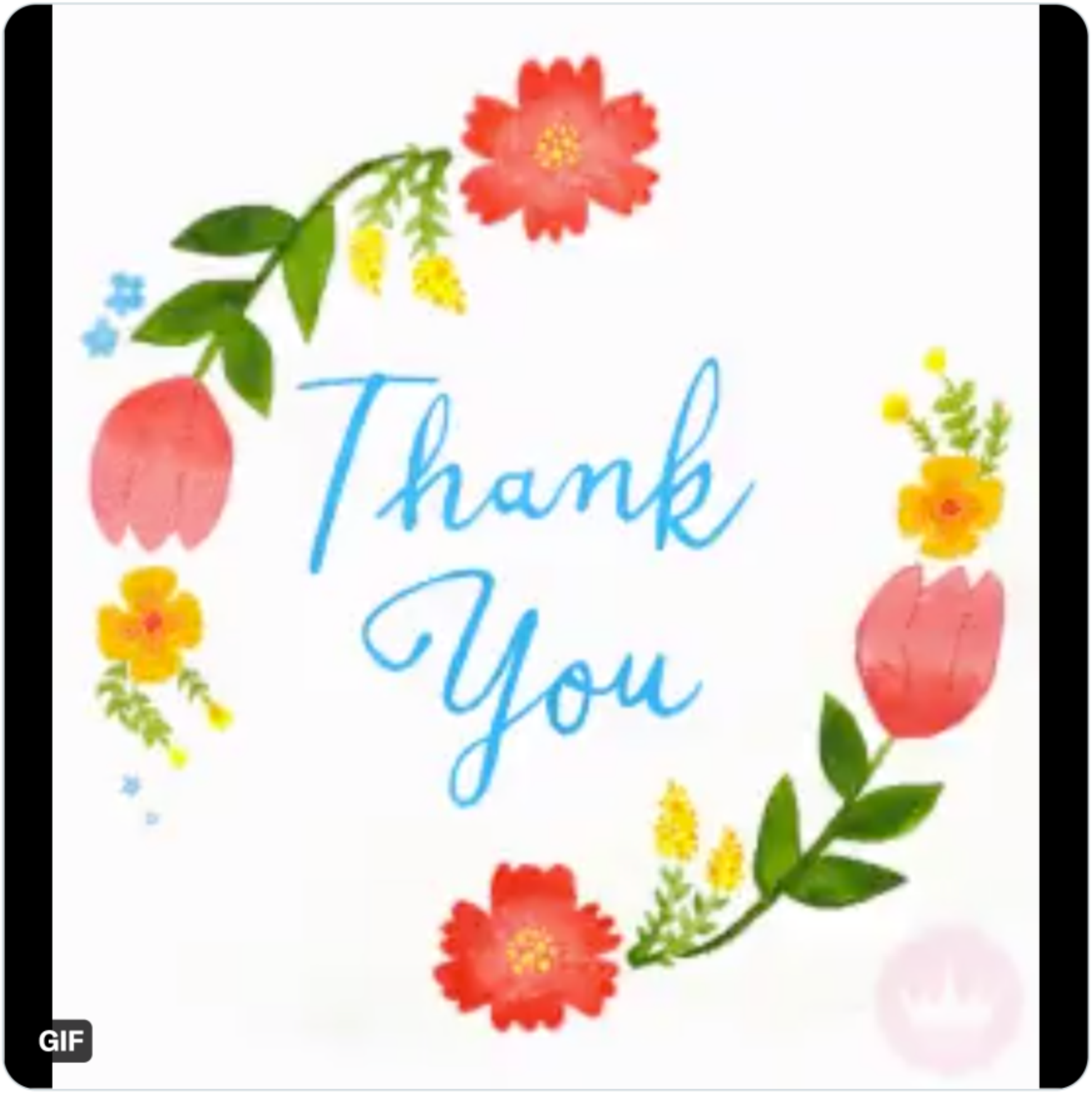

1

HashtagHPVtest @hashtaghpvtest · Feb 24

At your child's next doctors visit ask them about the HPV vaccine. 8/9

1

HashtagHPVtest @hashtaghpvtest · Feb 24

Thanks for following our account! We will be in touch soon! 9/9
